# BMPR2 promotes fatty acid oxidation and protects white adipocytes from cell death in mice

**DOI:** 10.1038/s42003-020-0928-y

**Published:** 2020-04-29

**Authors:** Shuwen Qian, Jiabao Pan, Yan Su, Yan Tang, Yina Wang, Ying Zou, Yaxin Zhao, Hong Ma, Youyou Zhang, Yang Liu, Liang Guo, Qi-qun Tang

**Affiliations:** 1 The Key Laboratory of Metabolism and Molecular Medicine of the Ministry of Education, Department of Biochemistry and Molecular Biology of School of Basic Medical Sciences, and Department of Endocrinology and Metabolism of Zhongshan Hospital, Fudan University, 200032 Shanghai, China; 2 Department of Orthopaedics, Shanghai Jiaotong University Affiliated Sixth People’s Hospital, 200032 Shanghai, China; 3 Center for Research on Reproduction & Women’s Health, University of Pennsylvania, Philadelphia, PA USA

**Keywords:** Apoptosis, Fatty acids

## Abstract

Adipocyte cell death is pathologically involved in both obesity and lipodystrophy. Inflammation and pro-inflammatory cytokines are generally regarded as inducers for adipocyte apoptosis, but whether some innate defects affect their susceptibility to cell death has not been extensively studied. Here, we found bone morphogenetic protein receptor type 2 (BMPR2) knockout adipocytes were prone to cell death, which involved both apoptosis and pyroptosis. BMPR2 deficiency in adipocytes inhibited phosphorylation of perilipin, a lipid-droplet-coating protein, and impaired lipolysis when stimulated by tumor necrosis factor (TNFα), which lead to failure of fatty acid oxidation and oxidative phosphorylation. In addition, impaired lipolysis was associated with mitochondria-mediated apoptosis and pyroptosis as well as elevated inflammation. These results suggest that BMPR2 is important for maintaining the functional integrity of adipocytes and their ability to survive when interacting with inflammatory factors, which may explain why adipocytes among individuals show discrepancy for death responses in inflammatory settings.

## Abstract (web summary)

Qian, Pan, Su et al. show that bone morphogenetic protein receptor type 2 (BMPR2) deficiency makes white adipocytes prone to death. This study suggests that BMPR2 is required for fat cells to effectively generate energy from the stored fat for their survival during energetically demanding situations such as inflammation.

## Introduction

Adipose tissues are important organs for metabolism. Both excessive accumulation of fat mass (obesity) and loss of fat (lipoatrophy) are detrimental to energy homeostasis. Adipose tissues are highly plastic not only because adipocytes can store (lipogenesis) or release fatty acids (lipolysis) readily upon stimuli, but also because adipocytes are continually renewed^1,2^. The number of fat cells is determined by the relative rates of adipogenic differentiation and adipocyte death. Although most reported studies focus on the adipocyte differentiation^3–5^, the significance of adipocyte death in maintaining structural and functional integrity of adipose tissue cannot be ignored.

Apoptosis is one common form of cell death. Adipocyte apoptosis is upregulated in human adipose tissue of patients with obesity and type 2 diabetes^6,7^. Moreover, low-grade inflammation with infiltration of macrophages and increased production of proinflammatory cytokine are associated with obesity^8–10^. The coincidence guides that apoptosis of adipocytes has been mostly studied in situations associated with inflammation. Dead adipocytes are surrounded by macrophages that form characteristic crown-like structures (CLS) around. The CLS have been described in adipose tissue from both obese mice and humans^11–13^. Although the upstream event remains unknown, apoptotic cell death of adipocytes is a key event that contributes to macrophage infiltration^6,12,14^, which in turn releases proapoptotic cytokines and poses direct effects on adipocytes^9,15,16^. Adipocyte apoptosis is also present in lipoatrophic adipose tissues. Long-term use of highly active antiretroviral treatment (HAART) in HIV-infected patients results in the appearance of peripheral fat wasting and central adiposity, the so-called HALS (HIV/HAART-associated lipodystrophy syndrome)^17,18^. Most data have shown adipocyte apoptosis in the lipoatrophic subcutaneous tissue of HALS patients. Lipodystrophy in patients with cancer cachexia is also accompanied with adipocyte apoptosis^19–21^. The association of adipocyte apoptosis with lipodystrophy suggests an important role for cell apoptosis in the progression of lipoatrophy, which is also supported by the study that targeted activation of caspase 8 in adipocytes induces apoptosis and causes mice to acquire the phenotype of lipoatrophy^22^.

Pyroptosis is another form of programmed cell death, which is characterized with inflammatory caspase 1 activation through the assembly of multiprotein complexes called inflammasomes. Activated caspase 1 induces pyroptosis and produces proinflammatory cytokines, such as interleukin-1β and interleukin-18 ^23,24^. It was reported that in leptin-deficient ob/ob mice, formation of active caspase 1 was detected in the cytoplasm of some hypertrophic adipocytes, indicating hypertrophic adipocytes likely induce obese adipocyte death by pyroptosis^25^.

A number of studies have associated proinflammatory cytokines with cell death. The most important cytokine is tumor necrosis factor α (TNFα). TNFα is secreted by immune cells or hypertrophic adipocytes^26^. The expression of TNFα in adipose tissue is elevated in a variety of experimental obesity models^27,28^ and obese humans^29–31^ as well as in patients with HALS and cancer cachexia^19,32,33^. TNFα is shown to induce or augment apoptosis in brown and white adipocytes ex vivo^34,35^. On the other hand, the absence of TNFα receptor results in a reduction in brown adipocyte apoptosis in genetically obese (ob/ob) mice^36^. However, TNFα gene polymorphisms have no association with the risk of HAART-associated lipid disorders in HIV patients^37,38^. The same phenomenon is observed in cancer cachexia patients, as microarrays from patient biopsies of subcutaneous white adipose tissue (WAT) showed no difference in TNFα mRNA and protein levels between control and weight-losing patients^39^. Such variation may be ascribed to host genotype. Genome-wide studies on HIV-infected patients suggest that genetic polymorphisms of genes involved in mitochondrial function and adipocyte metabolism are related to HALS^40,41^. These results suggest that structural and functional defect might make adipocytes more vulnerable to death in the context of elevated TNFα.

BMP (bone morphogenetic protein) signaling is important for the development and metabolism of adipose tissue. BMP7 promotes differentiation of brown preadipocytes^42^. BMP8B stimulates brown fat thermogenesis by both peripheral and central way^43^. BMP4 stimulates brown-like activity in white adipocyte^44^. BMP4 functions by binding to its receptors, which include both type I and type II receptors. BMPR2 is the type II receptor. Besides BMP4, other reported ligands of BMPR2 are BMP2, BMP5/6/7/8, BMP9/10, GDF5/6 (growth differentiation factor), and BMP15/GDF9^45^. The ligand assembles a heteromeric receptor complex for the activation of downstream cascades—the MAP kinase and Smad pathways^46,47^. Regarding the important roles of BMP4/7/8 in adipose development and metabolism, the level of their mutual receptor-BMPR2 might affect the structure and function of adipose tissue.

In the present study, we showed that BMPR2-deficient adipocytes were more susceptible to cell death, involving both pyroptosis and apoptosis. BMPR2 deficiency downregulated lipolysis by inhibiting the phosphorylation of perilipin. Decreased lipolysis caused the hypotrophy of adipocytes, which was associated with pyroptosis and activated inflammation. In addition, decreased lipolysis and low fatty acid β-oxidation (FAO) and oxidative phosphorylation (OXPHOS) activated mitochondria-mediated apoptosis when adipocytes were stimulated by TNFα. Thus, BMPR2 deficiency and failure of lipid metabolism drove adipocytes to enter into the vicious circle of inflammation and cell death. Our findings suggest BMPR2 could be a fundamental target for interfering with the survival and metabolism of adipocytes.

## Results

### BMPR2 knockout mice lose white fat during peri-weaning period

**Fig. 1:**
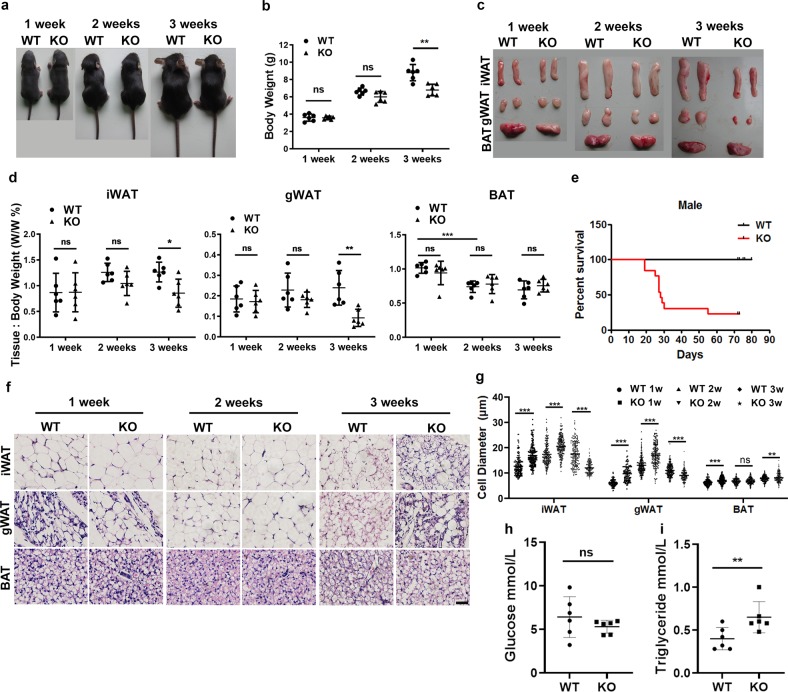
BMPR2 knockout mice lose white fat at weaning. **a** Representative pictures of WT and KO mice. **b** Body weight of WT and KO mice at different age (*n* = 6). **c** Representative pictures of WAT and BAT in WT and KO mice. **d** Fat index (percentage of fat pad weight to the whole body weight) of inguinal WAT (iWAT), gonadal WAT (gWAT), BAT in WT and KO mice (*n* = 6). **e** Survival curve of male WT and KO mice. **f** Representative images of H&E-stained sections of adipose tissues at different ages. Scale bar: 25 μm. **g** Quantification of adipocyte diameter of iWAT, gWAT and BAT from WT and KO mice. (Data were collected by using ImageJ software from H&E staining sections of three individual mice, five fields per mouse, and 10−15 cells per field in each group.) **h**, **i** Levels of glucose (**h**) and triglyceride (**i**) in serum of 2-week-old mice. Data in **b**, **d**, **g**, **h**, **i** are expressed as means ± SD. *p < 0.05, **p < 0.01, ***p < 0.001.

BMPR2 expressed in adipose tissue, and its amount was higher in mature adipocytes than in stromal vascular fraction (SVF) (Supplementary Fig. 1a). The expression pattern was similar in both mice (Supplementary Fig. 1a) and humans (Supplementary Fig. 1b). To find out the role of BMPR2 in the structure and function of adipocytes, BMPR2 were specifically knocked out in adipose tissue by crossing FABP4-cre transgenic and BMPR2^loxp/loxp^ mice. Mice bearing both FABP4-cre and BMPR2^loxp/loxp^ genotypes were designated as knockout (KO) mice, and with FABP4-cre and BMPR2^wt/wt^ genotypes were wild-type (WT) mice. Depletion of BMPR2 was verified by Western blot (Supplementary Fig. 1c) in isolated mature adipocytes of both inguinal WAT (iWAT) and BAT from 2-week-old mice. The mRNA levels of the two key transcription factors for adipogenesis (PPARγ and CEBPα) as well as the marker gene for mature adipocyte (adiponectin) did not significantly change in knockout mice (Supplementary Fig. 1d), suggesting BMPR2 knockout did not affect adipogenesis and formation of mature adipocytes. BMPR2 knockout mice appeared normal at birth, and the body weight (Fig. 1a, b) and fat mass index (Fig. 1c, d) showed no obvious difference from WT mice at the age of 1 week. However, the two parameters began to diverge at the age of 2 weeks, but they did not show a statistically significant difference between BMPR2 knockout and wild-type mice. At the age of 3 weeks, BMPR2 knockout mice lost a great amount of body weight and fat mass (Fig. 1a–d). The knockout mice began to die on day 19 postnatally, and about 70% could not survive to 4 weeks both for male mice (Fig. 1e) and female mice (Supplementary Fig. 1e). BMPR2 knockout mice retained little fat tissue when they died. The isolated fat tissue could not even float in the phosphate buffer solution (Supplementary Fig. 1f).

To study the detailed changes of adipose tissues, tissues were subjected to histological analysis. Although the amount of fat mass did not change significantly, the volume of individual adipocytes of both inguinal and gonadal fat was larger in BMPR2 knockout mice than in WT mice at the age of 1 and 2 weeks as shown by histological appearance and quantitation of cell diameters (Fig. 1f, g). However, at the age of 3 weeks, the average diameter of white adipocytes decreased, and BMPR2 knockout mice exhibited more cells with reduced size (Fig. 1f, g). The area covered by the adipocytes in white adipose tissues of BMPR2 knockout mice reduced, implying a breakdown of adipocytes. When the mice died, there was no visible adipocytes in white adipose tissue (Supplementary Fig. 1g). BMPR2 knockout had no effect on brown adipocytes in terms of fat index and histologically appearance (Fig. 1c, d, f, g).

In order to find out whether changes of adipocyte volume and fat mass had effects on energy homeostasis, levels of glucose and triglyceride in serum at the age of 19 days were measured. While glucose level showed no difference between the two types of mice (Fig. 1h), the triglyceride level of BMPR2 knockout mice was higher at 19 days than that of WT mice (Fig. 1i), which may result from the breakdown of BMPR2 knockout adipocytes. With regard to the fact that the fat loss is not necessarily the direct cause of mouse death, we also examined the histological alteration by H&E staining in some of the organs of the mice at the age of 19 days. Liver and kidney of BMPR2 knockout mice had no obvious changes as compared to those of WT mice. However, lungs of BMPR2 knockout mice showed thickened arterial wall (Supplementary Fig. 1h), which is characteristic of pulmonary arterial hypertension. Hearts of knockout mice were dilated (Supplementary Fig. 1h), which might be secondary to pulmonary hypertension. The pulmonary and heart failure might be involved in the death of BMPR2-knockout mice.

### Loss of white fat in BMPR2 knockout mice is associated with apoptosis and pyroptosis

**Fig. 2:**
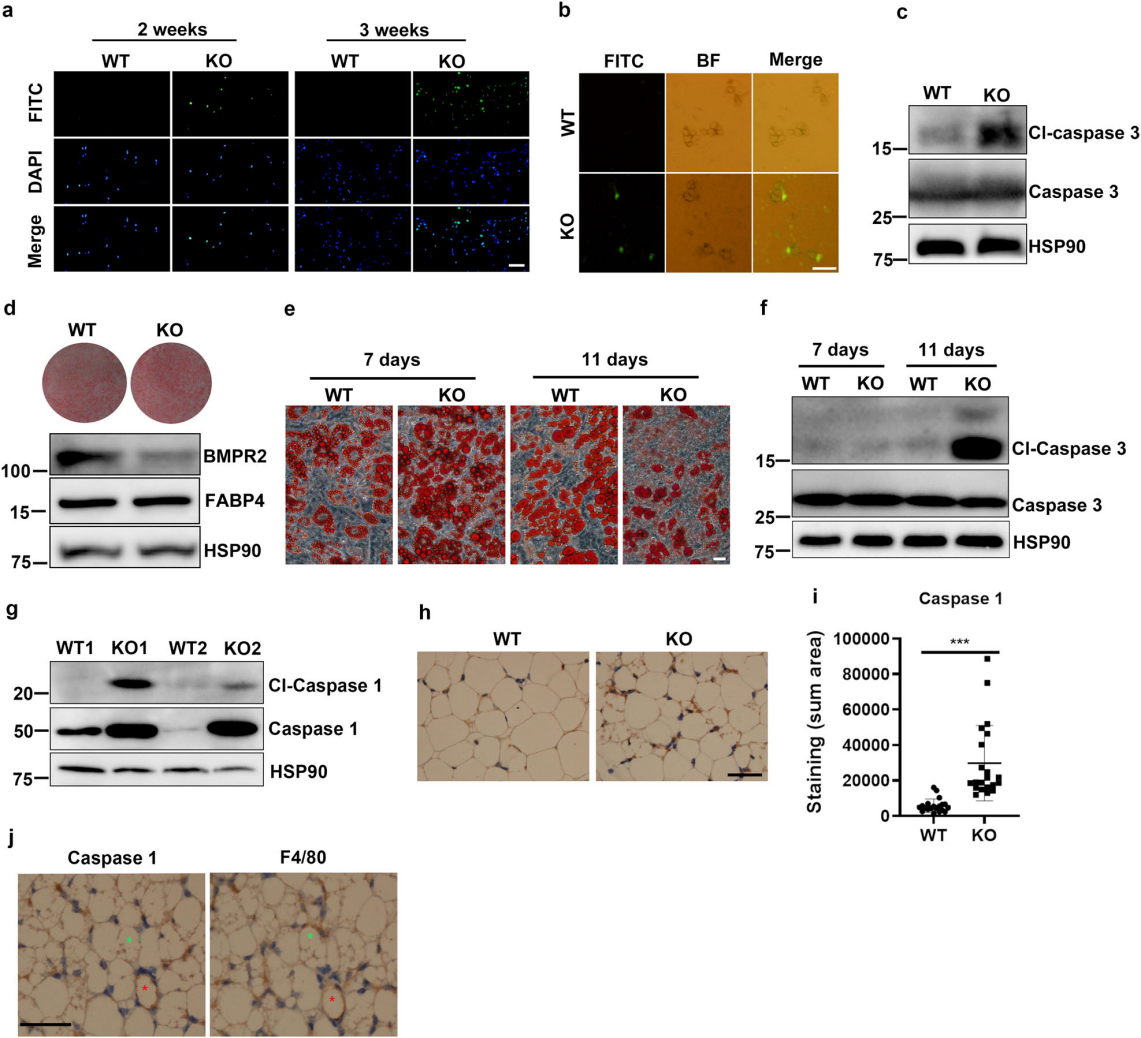
BMPR2 deficiency increases adipocyte death in white adipose tissue. **a** TUNEL (FITC) assay shows apoptosis in WT and BMPR2 knockout iWAT performed on tissue slides. Scale bar: 20 μm. **b** TUNEL (FITC) assay shows apoptosis in smeared mature adipocytes isolated from iWAT of 16-day-old mice. Scale bar: 20 μm. **c** Western blot analysis of caspase 3 in pooled mature adipocytes isolated from iWAT of four individuals in each group. **d** Precursor cells were isolated from iWAT of WT and KO mice and induced to differentiation. Oil red O staining for mature adipocytes showed the differentiation rate, and Western blots showed levels of BMPR2, FABP4, and HSP90 (loading control) in the two groups of cells. **e** Representative images showed the appearance of adipocytes of WT BMPR2 KO and cells at days 7 and 11 after induced to differentiation. Scale bar: 25 μm. **f** Western blot for cleaved caspase 3 showed the level of apoptosis of adipocytes in **e**. **g** Western blot analysis of caspase 1 in pooled mature adipocytes isolated from iWAT of four individuals in each group. **h** Immunochemistry showed positive staining for caspase 1 in iWAT of 19-day-old mice. Scale bar: 20 μm. **i** Quantitation of caspase 1-positive area on slides with ImageJ software (lower panel). Data were collected from sections of three individual mice, 6−8 fields per mouse. Data are expressed as means ± SD. ***p < 0.001. **j** Staining for caspase 1 and F4/80 by immunochemistry on sequential sections. Stars with the same color indicate one cell. Red star marked adipocyte expressed caspase 1 accompanied with F4/80 indicated crown-like structure (CLS). Green star marked adipocyte having CLS but was negative for caspase 1. Scale bar: 20 μm.

To find out whether white fat loss in BMPR2 knockout mice was associated with cell death, a terminal deoxynucleotidyl transferase (TdT) dUTP Nick-End Labeling (TUNEL) assay was firstly performed to evaluate apoptosis in subcutaneous white adipose of BMPR2 knockout and WT mice at 2 and 3 weeks of age, a time window when more collapsed adipocytes were appearing. A number of TUNEL-FITC-positive cells could be detected in the BMPR2 knockout mice at 2 weeks of age, which increased at 3 weeks (Fig. 2a). As the cell population in adipose tissue is heterogenous, to define the cellular specificity of apoptosis, mature adipocytes were isolated from 16-day-old mice and smeared for the TUNEL test. Positive staining was detected in BMPR2 knockout mature adipocytes (Fig. 2b). Western blot showed cleaved caspase 3 was consistently elevated in BMPR2 knockout mature adipocytes isolated from white adipose tissue (Fig. 2c). In contrast, TUNEL-positive adipocytes (Supplementary Fig. 2a) and cleaved caspase 3 (Supplementary Fig. 2b) were not detected in brown adipose tissue, which were consistent with the phenotype that BMPR2 mice did not lose brown adipose tissue (Fig. 1c, d).

The occurrence of apoptosis in BMPR2 knockout was further supported by ex vivo evidence. Preadipocytes were isolated from inguinal adipose tissue of both WT and BMPR2 knockout mice, cultured, and induced to adipogenic differentiation. Since expression of Cre recombinase is driven by FABP4, which is induced after differentiation, BMPR2 knockout did not alter the adipogenic differentiation as shown by the ratio of adipocytes stained with Oil red O and the level of FABP4 protein expressed in mature adipocytes (Fig. 2d, e). It is notable that the lipid droplets were larger in cells with lower levels of BMPR2 at day 7 after induction, as shown by the appearance of Oil red O-stained adipocytes (Fig. 2e). This is consistent with the phenotype of enlarged adipocytes in BMPR2 knockout adipose tissue (Fig. 1f, g). When allowed to grow till day 11, most of the cultured BMPR2 knockdown adipocytes broke down, leaving few complete adipocytes, but WT adipocytes maintained complete structure with larger lipid droplets than adipocytes at 7 days after differentiation (Fig. 2e). Levels of cleaved caspase 3 were extremely high in BMPR2 knockout cells at 11 days induction, but the control cells expressed no cleaved caspase 3 (Fig. 2f).

Besides the apoptosis, another form of cell death, pyroptosis, was also detected in BMPR2 knockout adipocytes. Western blot showed both caspase 1 and cleaved caspase 1 were increased in BMPR2-deficient adipocytes isolated from iWAT of 14-day-old mice (Fig. 2g). We also performed the immunohistochemistry (IHC) using anti-caspase 1 antibody. The results showed higher expression level of caspase 1 in BMPR2 knockout adipose tissue (Fig. 2h, i). The pyroptosis was supported by the RNA sequencing data that the NOD-like receptor signaling pathway, an important pathway regulating pyroptosis, was upregulated (Supplementary Fig. 3a–d). Meanwhile, we checked the localization of F4/80 in knockout iWAT on the sequential section, and found that caspase 1-expressed adipocytes were accompanied with F4/80 (crown-like structure, CLS). It was notable that F4/80-positive cells did not necessarily surround the caspase 1-positive adipocytes. There were cells with CLS that did not highly express caspase 1 (Fig. 2j), suggesting the existence of apoptotic cell death, which had been demonstrated in the BMPR2 knockout mice (Fig. 2a–c).

### BMPR2-deficient adipocytes produce a high level of inflammation and are susceptible to apoptosis

**Fig. 3:**
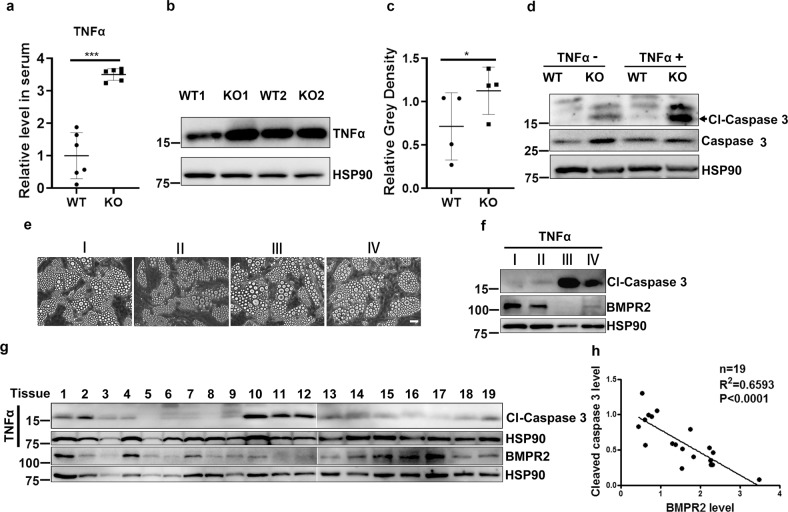
BMPR2-deficient adipocytes are sensitive to TNFα-induced apoptosis. **a** Levels of TNFα in serum of 14-day-old mice were determined using ELISA. *n* = 6. Data are expressed as means ± SD. ***p < 0.001. **b** Representative image of Western blot for TNFα in iWAT of two pairs of littermates at 14 days old. **c** Statistical analysis for the gray density of TNFα bands after normalized with HSP90 in Western blot assay described in **c** (*n* = 4). Data are expressed as means ± SD. *p < 0.05. **d** Western blot showed levels of cleaved caspase 3 and total caspase 3 in differentiated adipocytes from precursor cell in culture. **e** Image of mature adipocytes differentiated from isolated precursor cells in subcutaneous WAT at hip region of four human individuals. Scale bar: 25 μm. **f** Western blot showed levels of BMPR2 and cleaved caspase 3 (Cl-Caspase 3) in cells of **d**. **g** Western blot showing the levels of BMPR2 and cleaved caspase 3 in human subcutaneous adipose tissues from hip region (left panel). **h** Levels of BMPR2 and cleaved caspase 3 in **g** were normalized by HSP90 and analysis for linear regression to show their correlation (right panel).

Among the 884 genes upregulated in BMPR2 adipocytes group (Supplementary Fig. 3a, b), KEGG analysis recovered the TNF pathway (Supplementary Fig. 3c, d), involving genes coding for proinflammatory cytokines TNFα, IL1β, and IL6. We then measured the serum level of those cytokines in mice at 14 days old and found only the TNFα were increased (Fig. 3a), while IL1β and IL6 did not change (Supplementary Fig. 3e). Western blot showed the elevated level of TNFα in BMPR2 knockout iWAT (Fig. 3b, c), which derived from the hypertrophic adipocytes in BMPR2 knockout mice, as TNFα mRNA level was elevated in those cells (Supplementary Fig. 3f).

Due to the function of TNFα in inducing apoptosis of adipocytes, we wondered whether TNFα promote apoptosis of BMPR2 knockout adipocytes. Recombination TNFα was used to treat mature adipocyte differentiated from preadipocytes; Western blot showed much high level of cleaved caspase 3 in BMPR2 knockout cells after TNFα treatment (Fig. 3d). There was also a tracing level of cleaved caspase 3 with no exogenous TNFα stimulation (Fig. 3d), which may be due to endogenous TNFα released by adipocytes. To find out whether BMPR2 plays similar roles in affecting cellular apoptosis in human adipose tissue, human adipose tissue samples at the hip region were obtained, and preadipocytes were isolated and induced to adipogenic differentiation. Four lines of cells with adipogenic differentiation rates over 90% (Fig. 3e) were selected to minimize the bias caused by differentiation rate, and treated with recombinant human TNFα for 16 h. Cleaved caspase 3 was examined by Western blot to index the degree of apoptosis. Cells with lower levels of BMPR2 were more likely to express active caspase 3 (Fig. 3f). To expand the amount of samples, 19 individual samples of subcutaneous adipose tissue were minced into pieces of about 1 mm^3^ and treated with recombinant human TNFα for 16 h, followed by examination of cleaved caspase 3. Meanwhile, aliquots of tissue without TNFα treatment were preserved for the examination of BMPR2 expression. Results showed that levels of BMPR2 were negatively associated with that of cleaved caspase 3 (Fig. 3g, h). These results suggested that adipocytes with lower BMPR2 levels were more susceptible to TNFα-induced apoptosis. Local levels of TNFα were much lower in brown adipose tissue than in white adipose tissue when the mice were 3 weeks old (Supplementary Fig. 3g), which might be one possible reason that BMPR2-deficient brown adipose tissue showed no obvious apoptosis (Supplementary Fig. 2a, b).

### BMPR2 deficiency downregulates lipolysis and fatty acid OXPHOS

**Fig. 4:**
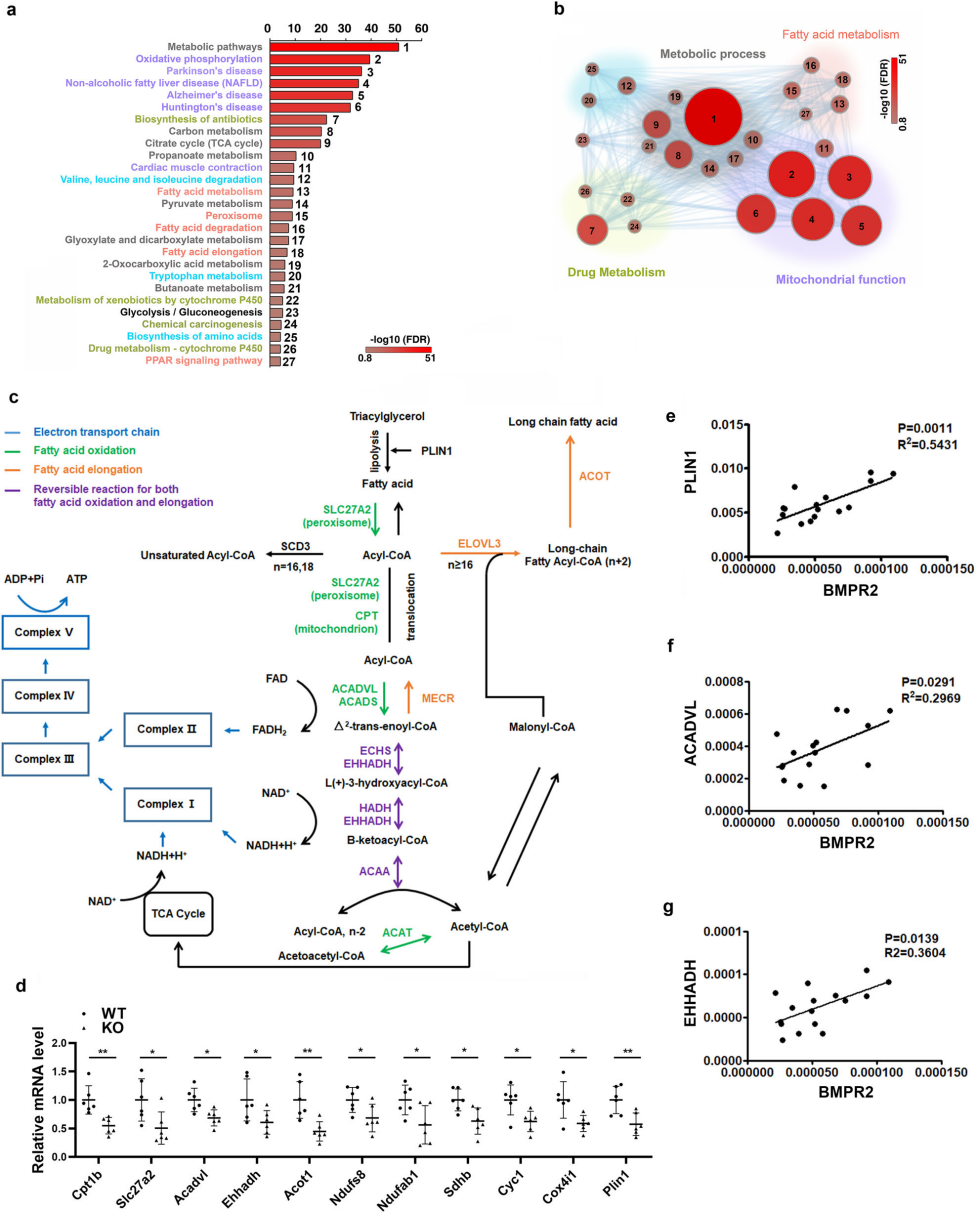
BMPR2-deficiency impairs fatty acid metabolism and oxidative phosphorylation. **a** Gene ontology analysis for downregulated genes in KEGG pathways, pathways with FDR < 0.1 and *p* value < 0.00015 were listed and grouped designated with different color. **b** Pathways with FDR < 0.1 and *p* value < 0.00015 were visualized in Cytoscape v.3.4.0, the node sizes and color presented *p* value and FDR, respectively. **c** Downregulated genes identified by sequencing involved fatty acid metabolism and oxidative phosphorylation. **d** Real-time PCR validation of genes selected from pathways in **c**. Total RNA were isolated from mature adipocyte isolated from inguinal fat (*n* = 6). Data are expressed as means ± SD. *p < 0.05, **p < 0.01. **e**−**g** Correlation between mRNA level of BMPR2 and indicated genes of human adipose tissue samples determined by quantitative PCR.

To find out why BMPR2 deficiency sensitizes white adipocyte to apoptosis, we analyzed 576 downregulated genes in the BMPR2 adipocytes group (Supplementary Fig. 3a, b). Gene ontology analysis in KEGG pathways showed all of the downregulated genes were involved in metabolism, among which there were 27 pathways with FDR < 0.1. The most striking pathway was for oxidative phosphorylation, involving genes belonging to Complex I-V of electron transport chain (Fig. 4a, b and Supplementary Table 1), indicating mitochondrial dysfunction may play an important role in apoptosis of BMPR2-deficient adipocytes. Another group of pathways was those for fatty acid metabolism (Fig. 4a, b and Supplementary Table 1). After analyzing these genes included in the fatty acid metabolism, we found that they regulated fatty acid oxidation, elongation, and biosynthesis of unsaturated fatty acid (Fig. 4c). Notably, these genes catalyze reaction using acyl-CoA or free fatty acid as substrates, suggesting that the shortage of acyl-CoA or free fatty acid accounts for the changed fatty acid metabolism. We found in PPAR signaling pathway, in addition to the genes regulating fatty acid β-oxidation, *Plin1* was also included (Fig. 4c and Supplementary Table 1). *Plin1* encodes perilipin, one lipid droplet coating protein hydrating triacylglycerol to free fatty acid, which is the energy source for oxidative phosphorylation. Selective genes for fatty lipid metabolism, electron transport chain as well as *Plin1* were confirmed by quantitative PCR (Fig. 4d). The inhibitive expression of *Plin1* was through the smad pathway rather than the P38 pathway, as the knockdown of smad4 in cultured adipocytes leads to downregulation of *Plin1* (Supplementary Fig. 4a); however, knockdown of p38 unexpectedly upregulated the *Plin1* mRNA level (Supplementary Fig. 4b). Genes for fatty acid transportation (CD36) and synthesis (*Fasn*, *Acc*) did not change (Supplementary Fig. 4c). We also analyzed the expression levels of BMPR2 and some genes from the sequence data in human subcutaneous adipose tissue and found BMPR2 was positively related with *PLIN1* (Fig. 4e), *ACADVL* (Fig. 4f), and *EHHADH* (Fig. 4g).

### BMPR2 deficiency impairs regulation of perilipin upon TNFα stimulation

**Fig. 5:**
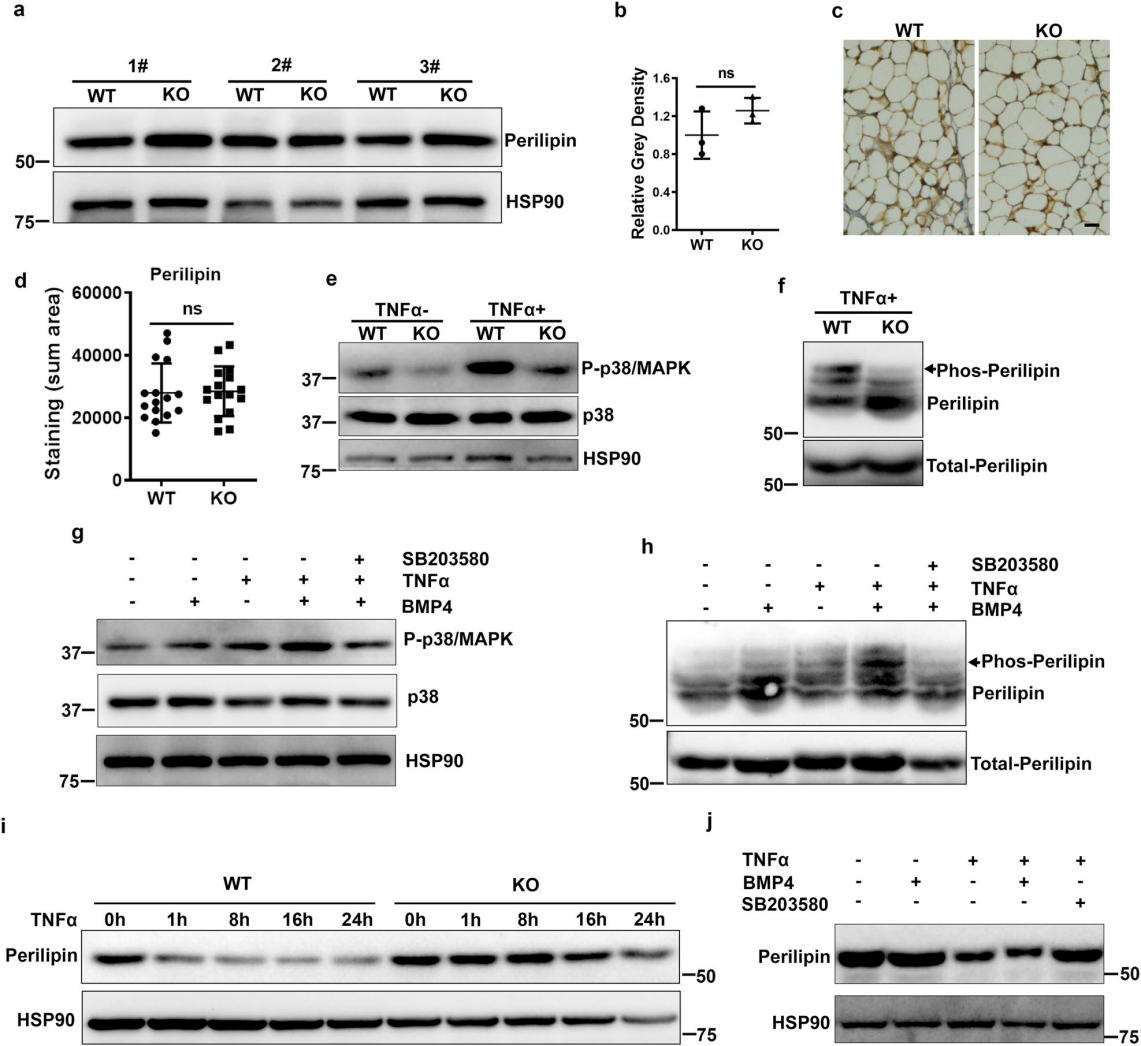
Dysregulation of perilipin in BMPR2 knockout adipocytes. **a** Western blot for perilipin in mature adipocytes isolated from iWAT of BMPR2 knockout mice and their littermates (1#, 2#, 3# represents three different litters). **b** Relative gray intensity of the bands normalized with HSP90 was quantitated using ImageJ software. **c** Representative image of IHC staining for perilipin on iWAT sections. Scale bar: 20 μm. **d** The positive staining area of perilipin in **c** was quantitated using ImageJ software. Data were collected from sections of three individual mice, 5–6 fields per mouse. **e** Western blot showed phosphorylated p38/MAPK and total p38 in adipocytes differentiated from precursor cells with or without 20 μg/ml TNFα treatment for 1 h. **f** The lysate of cultured cells was separated by a phosphate affinity SDS-page and blotting for mobility shift detection of phosphorylated perilipin. The arrow head pointed bands were those affected by BMPR2 level. **g** Western blot showed phosphorylated p38/MAPK and total p38 in adipocytes differentiated from precursor cells after treatment with 20 μg/ml TNFα with or without 10 ng/ml BMP4 for 1 h. SB203580 (10 μM) was used to pretreat cells for 1 h to inhibit P-p38. **h** Phosphorylated perilipin in cells referring to **g** was examined using the method indicated in **f**. The arrow head pointed bands were those affected by BMP4 activation and p38 inhibition. **i** Differentiated mature adipocytes in culture were treated with 20 μg/ml mouse recombinant TNFα; perilipin level along time course was shown by Western blot. **j** Differentiated mature adipocytes in culture were treated with 20 μg/ml TNFα in the presence or absence of 10 ng/ml BMP4 and P-p38 inhibitor SB203580 (10 μM) for 16 h, perilipin was shown by Western blot. Data in **b** and **d** are expressed as means ± SD. ns represents non-significant.

BMP signaling seemed to promote transcription of *Plin1* (Fig. 4d, Supplementary Fig. 5a). Regarding perilipin functions in the regulation of lipolysis, the decreased *Plin1* (Fig. 4d) and unchanged *Plin2* and *Plin3* (Supplementary Fig. 5b) mRNA levels in BMPR2 knockout adipocytes may contribute to the larger lipid droplet phenotye (Figs. 1f and 2e). However, perilipin protein levels in BMPR2 knockout and WT mice were almost the same as determined by Western blot (Fig. 5a, b). Immunohistochemical staining of perilipin also showed similar protein levels (Fig. 5c, d). We found that perilipin was dynamically decreased when the adipocytes were induced to lipolysis (Supplementary Fig. 5c), implying that factors involved in perilipin will affect its lipolysis function. BMPR2 knockout had no effect on isoproterenol-stimulated lipolysis (Supplementary Fig. 5d) and downregulation of protein level of perilipin (Supplementary Fig. 5e).

Upon binding with its ligands (BMP4/BMP7/BMP8/BMP10), BMPR2 activates the downstream molecule p38/MAPK by phosphorylation, and P-p38/MAPK acts as a kinase to phosphorylate its targets. As expected, we found that levels of P-p38/MAPK were reduced in differentiated BMPR2 knockout adipocytes in culture (Fig. 5e). TNFα can elevate the level of P-p38/MAPK, which was attenuated by BMPR2 knockdown (Fig. 5e). At the same time, the phosphorylation of perilipin was decreased, as indicated by less dense bands binding to a Phos-tag (Wako) that captures phosphomonoeater dianions (Fig. 5f). Consistently, BMP4 treatment enhanced TNFα-stimulated activation of p38/MAPK (Fig. 5g) and phosphorylation of perilipin (Fig. 5h), both of which were inhibited by the P-p38/MAPK inhibitor SB203580 (Fig. 5g, h). The dynamic change of perilipin was impaired in differentiated adipocytes, as Western blot showed degradation of perilipin after TNFα stimulation was impaired in BMPR2 knockout adipocytes (Fig. 5i). Regulation of perilipin after TNFα treatment was also evidenced in BMP4-treated cells, as BMP4 enhanced TNFα-stimulated degradation, which was blocked by the P-p38/MAPK inhibitor SB203580 (Fig. 5j). There was a shift of the perilipin band after TNFα stimulation, which was enhanced by BMP4 (Fig. 5j), supporting the idea that activation of BMPR2 phosphorylated perilipin. TNFα-induced changes of perilipin in adipose tissue were the same if the tissue was incubated with TNFα ex vivo. After treatment with TNFα for 16 h, perilipin in WT tissue was downregulated, but that in knockout tissue was retained at a relatively high level (Supplementary Fig. 5f). These results demonstrated that activation of BMPR2 influenced the regulation of perilipin through phosphorylation.

### Lipolysis inhibition and energy shortage induce cell death of adipocytes

**Fig. 6:**
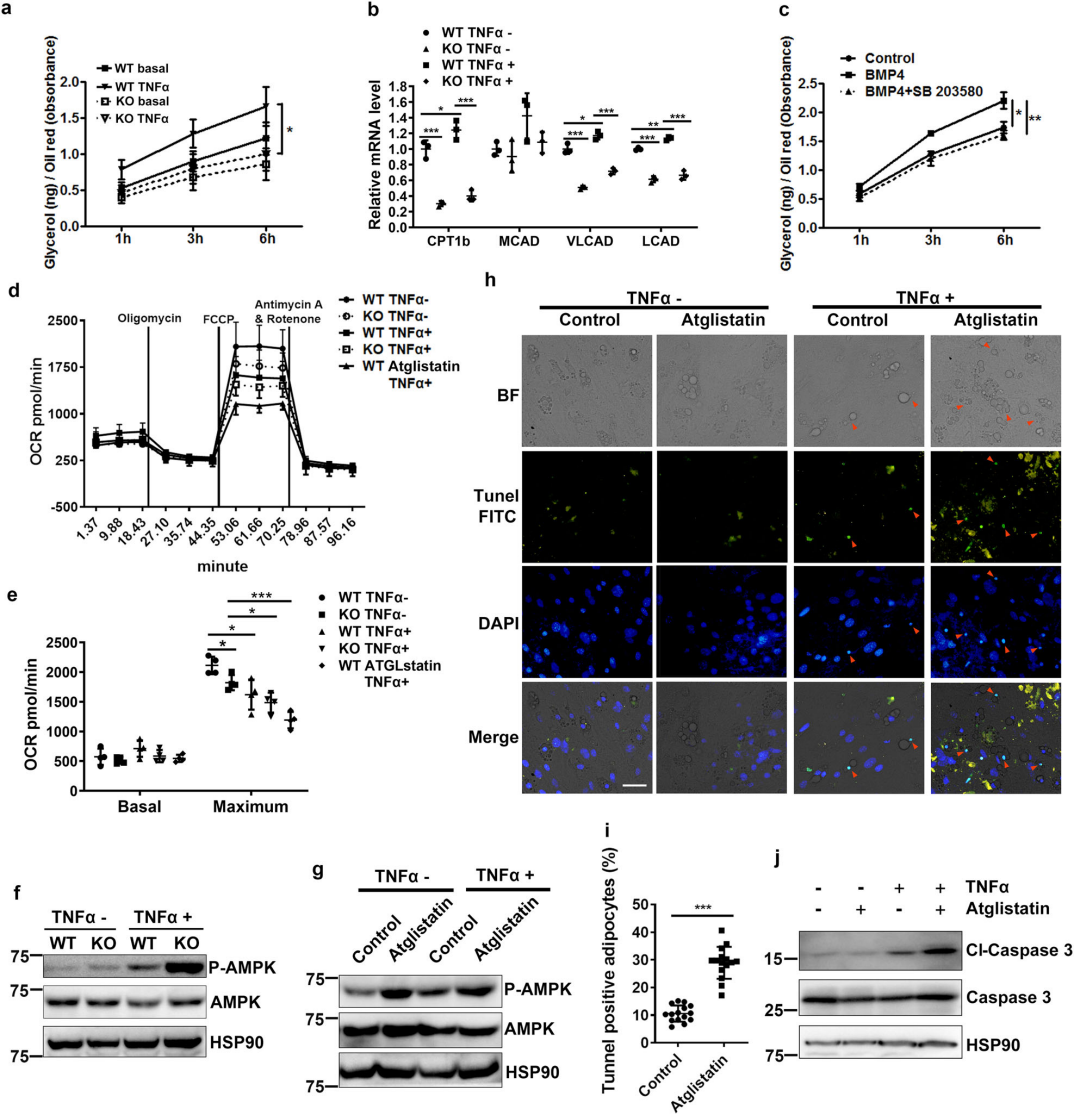
Reduced lipolysis induces apoptosis through mitochondrial pathway. **a** Glycerol in culture medium of adipocytes was determined by colorimetry and normalized to total amount of neutral lipid (stained Oil red O rinsed with methanol and absorbance measured at 490 nm). **b** Quantitative PCR determined expression of genes for fatty acid oxidation in cultured adipocytes differentiated from SVF, which were treated with 20 μg/ml TNFα for 1 h. **c** Adipocytes were treated with BMP4 or with P-p38 inhibitor SB283058 pretreated and subjected to 20 μg/ml TNFα-stimulated lipolysis; glycerol in culture medium was determined. **d** Adipocytes in culture were treated with 20 ng/ml TNFα for 16 h; OCR was determined using a seahorse XF24 analyzer. Data were from four replications and expressed as mean ± SD. **e** Quantification of data in **d**. Data were expressed as means ± SD. *p < 0.05, **p < 0.01, ***p < 0.001. **f**, **g** Representative Western blot determined the level of phosphorylated AMPK. Adipocytes were treated with 20 ng/ml TNFα for 16 h. Atglistatin (20 μM) pretreated cells to inhibit lipolysis. **h**, **i** TUNEL staining (FITC positive) of differentiated adipocytes treated and stimulated in the presence and absence of Atglistatin to show apoptotic cells (**h**). Scale bar: 20 μm. Ratio of TUNEL-positive cells was quantified (**i**). Data were from three replications, amounting to 15 microscopic fields in each group. **j** Western blot shows the level of caspase 3 in cultured adipocytes of **h**.

Phosphorylation of perilipin is an indispensible process for lipolysis. To explore whether defects in perilipin phosphorylation interrupt lipolysis in BMPR2 knockout adipocytes, the amount of glycerol released into the medium was determined with a colorimetric method. TNFα-induced glycerol release was much lower for BMPR2 knockout adipocytes than control adipocytes (Fig. 6a). The fatty acid oxidation of knockout adipocyte was consequently decreased in both 1-h-TNFα-treated and untreated groups (Fig. 6b). BMP4 treatment enhanced TNFα-stimulated glycerol release from differentiated adipocytes in culture, which was blocked by the P-p38 inhibitor SB203580 (Fig. 6c). These results suggested that BMP signaling through BMPR2 maintains the regulation of perilipin and guarantees lipolysis of adipocytes.

To explore the consequence of inhibited lipolysis on the metabolic function of adipocytes, analysis with a Seahorse XF24 analyzer (Agilent) was performed to detect mitochondrial energy consumption in differentiated adipocytes after treatment with TNFα for 16 h. BMPR2 knockout adipocytes generally had lowered oxygen consumption rates (OCR) as compared to WT adipocytes although the decrease was not statistically significant (Fig. 6d, e). After TNFα treatment, the basal OCR was elevated to a small extent, while the maximum OCR was decreased, suggesting that fatty acid depletion after chronic stimulation to lipolysis impaired mitochondrial respiratory capacity. BMPR2 knockout adipocytes showed even lower maximum OCR than WT adipocytes due to the inhibition of lipolysis by BMPR2 knockout (Fig. 6d, e).The decrease in maximum OCR when lipolysis was inhibited was also supported by the outcome of cells treated with Atglistatin, an inhibitor of lipolysis through adipose triglyceride lipase (ATGL) (Fig. 6d, e). In addition, BMPR2 knockout decreased oxidation of exogenous fatty acid in adipocytes (Supplementary Fig. 6a). Altogether, these results supported the gene expression data from RNA-sequence. It is known that the AMP-activated protein kinase (AMPK), a ubiquitous sensor of cellular energy, is phosphorylated and activated if an energy deficit is detected. Therefore, levels of P-AMPK were examined in BMPR2 knockout adipocytes as well as in Atglistatin-treated adipocytes. After treatment with TNFα for 16 h, adipocytes showed an elevated amount of P-AMPK, which was enhanced in both BMPR2 knockout (Fig. 6f) and Atglistatin-treated adipocytes (Fig. 6g), suggesting a shortage of energy in these cells.

To verify whether inhibition of lipolysis induced apoptosis upon TNFα treatment, we examined apoptosis in cultured adipocyte treated with Atglistatin. Differentiated adipocytes from precursor cells isolated from subcutaneous adipose tissue were treated with Atglistatin before the addition of TNFα, and TUNEL assay was performed to detect apoptosis. TNFα-induced apoptosis of mature adipocytes was shown by the condensed TUNEL-FITC-positive nuclei alongside lipid droplets in adipocytes (Fig. 6h), with a percentage of about 10% (Fig. 6i). With the addition of Atglistatin, the percentage of apoptotic adipocytes increased to around 30% (Fig. 6h, i). Along with the apoptotic phenotype, those adipocytes were increased with levels of cleaved caspase 3 (Fig. 6j). The results also support the increased levels of cleaved caspase 3 in BMPR2 knockout adipocytes as shown in Fig. 3d. There was a great amount of cleaved caspase 9 in BMPR2- knockout adipocyte after induction with TNFα (Supplementary Fig. 6b), indicating the mitochondrial pathway was involved in apoptosis. Consistently, cytochrome C in the cytosol was elevated in BMPR2 knockout adipocytes (Supplementary Fig. 6c). These results suggest inhibition of lipolysis in BMPR2 triggers apoptosis through mitochondrial pathway.

## Discussion

**Fig. 7:**
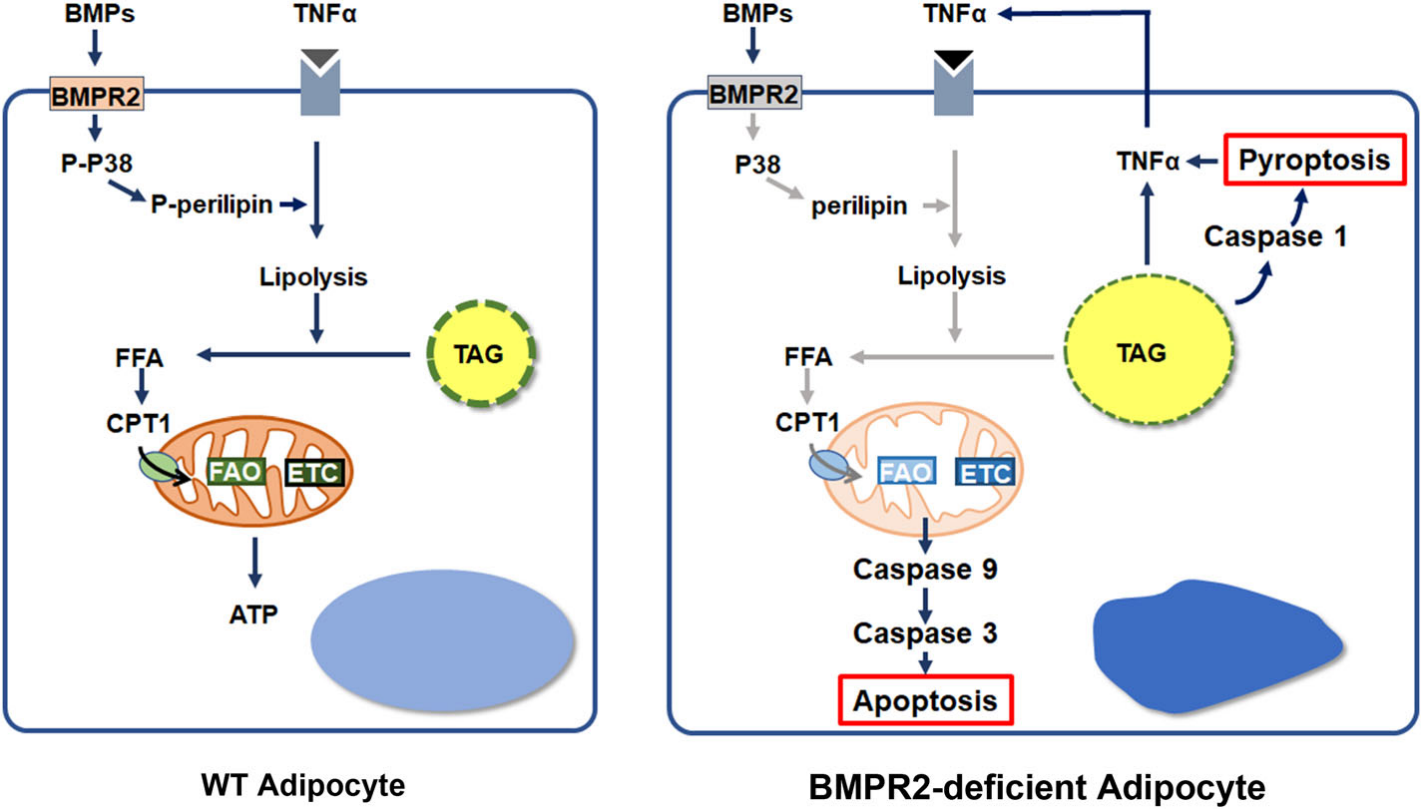
Schematic model of the role of BMPR2 in the inhibition of fatty acid metabolism and induction of cell death. BMPR2 signaling facilitates lipolysis by activating phosphorylation and dynamics of perilipin, thus guaranteeing the availability of fatty acid for β-oxidation (FAO) and oxidative phosphorylation (OXPHOS) through electron transport chain (ETC). BMPR2 deficiency disrupts this action, leading to the failure of OXPHOS, and activates mitochondria-mediated apoptosis in adipocytes when adipocytes were stimulated by TNFα. Meanwhile, deficiency of lipolysis leads to hypertrophy of adipocytes and subsequent pyroptosis and inflammation, among which elevation of TNFα is prominent. TNFα in turn poses more pressure on adipocyte, accelerating the process of cell death. BMPR2 deficiency thus renders the white adipocytes vulnerable to cell death. FFA free fatty acid, TAG triacylglyceride.

Adipose tissues are highly dynamic organs, and readily remodel upon stimulation, such as nutrient intakes, cold stimulation, and physical activity. Accordingly, processes such as cell differentiation and death, lipid synthesis and lipolysis, synthesis of adipokines, immune response and inflammation are taking place to adapt the stimuli and maintain energy homeostasis^48^. For example, adipocyte hypertrophy comes up with more space for storage of these nutrients in the form of triglyceride. The lipid load induces the synthesis and secretion of cytokines such as TNFα, interleukins (ILs) and C-C motif chemokine ligands (CCLs), which stimulated inflammation. Temperate inflammation is essential for maintaining the normal function of adipose tissue. Inflammation that accompanies the activation of macrophages has a function in tissue remodeling, such as promoting the differentiation of preadipocytes^49,50^. In addition, inflammatory cytokines such as TNFα and IL-6 increase basal respiration of adipocytes^51^. They both induce lipolysis, and the free fatty acids are used by mitochondrial oxidative phosphorylation to produce ATP, which links inflammation with energy metabolism^52^. In tissues with higher metabolic activities such as liver and adipose tissue, for example, hepatocytes and adipocytes are associated with Kupffer cells and macrophages^53,54^. Lymph nodes are always covered by perinodal adipose tissue, which provides energy for immune responses^55^. However, overactivated inflammation causes a series of pathological alteration. Proinflammatory cytokines are thought to promote apoptosis of adipocyte, among which TNFα is regarded as culprit. Although there is evidence from in vitro experiments that TNFα induces adipocyte apoptosis^34,35^, the association of high TNFα levels and cell death in human adipose tissue is controversial. Patients with HALS and cancer cachexia show no difference for TNFα mRNA level in white- adipose tissue between control and lipodystrophy patients^37–39^. The discrepancy indicates some uncovered mechanism involved in the death of adipocyte. In the present study, BMPR2 deficiency disrupted phosphorylation and dynamics of perilipin, which decreased lipolysis of white adipocytes. The impaired lipolysis, on one hand, led to the lipid overload, thus stimulating the pyroptosis and inflammation with TNFα synthesis and release. On the other hand, inhibited lipolysis caused a shortage of energy supplies for mitochondrial oxidative phosphorylation when adipocytes were stimulated by TNFα, and activated mitochondria-mediated apoptosis (Fig. 7). These results suggest that BMPR2 knockout enhances cell death in ways of both pyroptosis and TNFα-induced apoptosis, which is also through the intrinsic mitochondrial apoptotic pathway.

Perilipin is the most abundant protein associated with lipid droplets of adipocytes and functions to control both basal and stimulated lipolysis^56^. Perilipin protects intracellular lipid droplets from neutral lipases within the cell under basal conditions. In response to lipolytic stimuli, perilipin is phosphorylated on several serine residues and recruits hormone-sensitive lipase and other lipases to the surface of the lipid droplet to break down lipid. Here we found that TNFα-activated perilipin phosphorylation is indicated by shifted bands (Fig. 5j and Supplementary Fig. 5f), which can bind to a Phos-tag and lagged on Western blot (Fig. 5h); the appearance of these bands was inhibited by the P-p38/MAPK inhibitor SB203580 (Fig. 5h and j). Since downstream signaling of BMPR2 involves p38/MAPK, TNFα-stimulated perilipin phosphorylation was hampered by depletion of BMPR2 (Fig. 5f). It seems that TNFα stimulates perilipin phosphorylation at different residues from isoproterenol, which activated PKA, because BMPR2 knockout has no effect on isoproterenol-stimulated lipolysis (Supplementary Fig. 5d). Although the detailed dynamics of how perilipin is processed after the breakdown of lipid droplets is unknown, several studies have also shown downregulation of endogenous perilipin levels^57,58^. In addition to regulation of perilipin at the protein level through phosphorylation, BMP signaling apparently regulates perilipin at the transcriptional level, for BMPR2 knockout decreased perilipin mRNA levels in adipocytes (Fig. 4d). Consistently, BMP4 overexpression in adipocytes led to increased levels of perilipin (Supplementary Fig. 5a). The transcriptional regulation of perilipin by BMP signaling is through Smad pathway rather than the P38/MAPK pathway (Supplementary Fig. 4a, b). The high levels of perilipin in adipocytes induced by BMP4 may make lipid droplets more sensitive to lipolysis, and readily consume fatty acid, which may explain why BMP4 transgenic mice have a lowered serum triglyceride level and are metabolically improved, as shown in our previous study^44^. The correlation between BMP4 and lipid metabolism is also supported by the analysis of single nucleotide polymorphisms within the BMP4 region in 6822 participants and finding rs8014363 has a statistically significant association with triglyceride level^59^.

The correlation between inhibited lipolysis and cell death is also indicated by hormone-sensitive lipase (HSL) knockout mice, a model of adipocyte hypertrophy without obesity. HSL null mice show reduced total WAT mass as compared to control on a chow diet, and this difference becomes more obvious on a high fat diet or leptin deficiency (ob/ob)^60,61^. Histological changes exhibit increased heterogeneity of cell size, with both larger and smaller adipocytes. Another research shows there is a 15-fold increase in adipocyte death and formation of macrophage syncytia, coincident with increased tumor necrosis factor gene expression in WAT of HSL mice^11^. These data suggest the hypertrophy of adipocytes resulting from HSL knockout may induce cell death along with inflammatory reaction. The inflammation thus in turn accelerates the cell death indicated by a marked increase in small, lipid-devoid cells when combining with leptin deficiency^60^, which is regarded to lead to a higher level of inflammation in adipocytes. It is very interesting that in WAT of HSL null mice, perilipin immunohistochemistry demonstrating CLS selectively form around perilipin-free adipocyte-like structures but not around viable adipocytes expressing perilipin. The changes in WAT of HSL-null mice agree with those of BMPR2-knockout mice. In our present study, some of the changes of BMPR2 null adipocyte, such as the increased sensitivity to cell death and phosphorylation of AMPK in the presence of TNFα, were simulated by inhibition of ATGL. Collectively, these results suggest that with regard to influencing cell survival in TNFα-activated scenario, ATGL and HSL perform similarly. It is noted that inhibition of ATGL increased phosphorylation of AMPK even in the absence of TNFα (Fig. 6g), which implies that ATGL may have some unidentified function related to energy metabolism. However, there has been no data about whether inhibition of HSL has the same effect on phosphorylation of AMPK. So whether ATGL has different energetic functions from HSL need more comprehensive studies.

BMPR2 knockout induced both apoptosis and pyroptosis in adipocyte. The pyroptosis was indicated by the high expression of caspase 1 in WAT of BMPR2-knockout mice shown by IHC (Fig. 2h, i), with macrophages surrounding to form CLS shown by the sequential section (Fig. 2j). However, not all of the CLS accompany the caspase 1 high-expressing adipocytes (Fig. 2j), suggesting the presence of apoptosis, which was evidenced by the TUNEL staining (Fig. 2a, b). BMPR2 knockout stimulates genes for TNF pathway (Supplementary Fig. 3c, d) and increases TNFα expression and release from adipocytes (Supplementary Fig. 3f, Fig. 3a) to pose the apoptotic effects on adipocytes. The increased inflammatory response might result from reduced lipolysis and adipocyte hypertrophy, but there is also a possibility that BMPR2 signaling directly regulates the inflammation through signaling such as the NF-kappa B signaling pathway (Supplementary Fig. 3c, d).

Although BMPR2 was knocked down in macrophages due to the knockout model that was driven by FABP4 cre, the knockdown has no obvious effects on the percentage of M1 macrophage exhibited by our previous report^62^. Moreover, BMPR2 knockout specifically in macrophages driven by lysz-cre has no reported mouse death, suggesting that the macrophage regulation does not contribute to the phenotype of mouse death in the present mouse model that has a lower survival rate during weaning (Fig. 1e and Supplementary Fig. 1e). We suppose that the dysfunction of lung and heart (Supplementary Fig. 1h) might be the direct cause of mouse death. Although the less fat mass would not definitely cause mouse death, the high levels of inflammation associated with pyroptosis and apoptosis in BMPR2 knockout mice may contribute to the dysfunction of lung, as TNFα drives pulmonary arterial hypertension by suppressing the BMP type-II receptor^63^.

In summary, BMPR2 knockout adipocytes fail in lipid metabolism and render white adipocytes vulnerable to cell death, which involves both pyroptosis and apoptosis. These results suggest that structural and functional defects in adipocytes caused by genetic variant such as BMPR2 mutant may determine the fate of adipocytes when faced with the nutrients overload and consequent inflammatory pressure. Our findings imply a previously unidentified, potential target for treating metabolic disorders.

## Methods

### Human adipose tissue samples

Subcutaneous adipose tissue at hip region were obtained from patients who underwent plastic surgery irrelevant to metabolic disease in Shanghai Jiaotong University Affiliated Sixth People’s Hospital. They had no diagnostic metabolic disorders. Samples were obtained from male and female patients between the ages of 25 and 60 years. Samples were kept on ice after dissection for no more than 2 h before proceeding to cultural procedure. This study was approved by the ethics committees of Shanghai Medical College, Fudan University and was in accordance with the principle of the Helsinki Declaration II. Written informed consent was obtained from each participant.

### Generation of adipose tissue-specific BMPR2 knockout mice

To generate mice with an adipocyte-specific knockout of BMPR2, BMPR2LoxP/LoxP (generously provided by En Li, Cardiovascular Research Center, Massachusetts General Hospital, Harvard Medical School) were crossed with mice expressing Cre recombinase under the control of the adipocyte-specific promoter FABP4 (Jackson Laboratory). Genotyping was performed by PCR. Studies were performed in FABP4-Cre-BMP4LoxP/LoxP mice (KO) and littermate controls lacking the LoxP sites—Fabp4-cre-BMP4+/+ (WT). Mice were maintained under 12-h light/12-h dark cycles with unlimited access to food and water. All studies were approved by the Animal Care and Use Committee of Shanghai Medical College, Fudan University.

### Isolation of SVF and adipocytes from adipose tissue

Adipose tissue was harvested and the SVF cells isolated by enzymatic digestion (collagenase VIII; Sigma). The digested tissue was filtered through a 100 μm mesh filter to remove debris and centrifuged. The adipocytes were floating above the supernatant. The cellular pellet involving SVF was re-suspended with an ammonium chloride lysis buffer to remove red blood cells.

### Induction of adipogenesis from precursors in iWAT

Preadipocytes were isolated from iWAT of mice, plated at low density, and cultured in DMEM/F12 containing 10% calf serum. Two-day postconfluent (designated day 0), cells were induced to differentiate with DMEM containing 10% (vol/vol) fetal bovine serum (FBS), 10 μg/ml insulin (I), 1 μM dexamethasone (D), 0.5 mM 3-isobutyl-1-methyl-xanthine (M) and 1 μM rosiglitazone until day 2. Cells were then fed with DMEM/F12 supplemented with 10% FBS and 10 μg/ml insulin and 1 μM rosiglitazone for 1 day followed by medium with insulin for 2 days, after which they were fed with DMEM/F12 containing 10% FBS for 2 more days. The differentiated mature adipocytes were then subjected to cytokine treatment by adding to the medium.

### Tissue culture

Human subcutaneous adipose tissues from hip region were minced into pieces of about 1 mm^3^ and treated with human recombinative TNFα at a concentration of 50 ng/ml for 16 h.

### Hematoxylin-and-eosin staining

Standard hematoxylin and eosin (H&E) staining were performed on 5 μm paraffin sections of white adipose tissue and interscapular brown adipose tissue. Cell diameters were measured with ImageJ from the H&E staining section of three individual samples in each group.

### Immunohistochemistry and TUNEL assay

Inguinal adipose tissue was excised, fixed with 4% paraformaldehyde overnight, embedded in paraffin and cut into 4-μm-thick sections. Immunohistochemistry was performed according to the VectaStain Elite ABC kit protocols (Vector Laboratories). The antibodies were anti-F4/80 (Abcam: ab6640) and anti-Caspase 1 (Abcam: ab138483). The area of positive staining was quantified using image-pro plus software.

For TUNEL assay, sections of adipose tissue, cultured mature adipocytes, or smeared adipocytes isolated from adipose tissue were subjected to examination according to the manufacturer’s instruction (In Situ Cell Death Detection Kit, Roche 11684795910).

### RNA sequencing of WT and BMPR2 knockout adipocytes

Total RNA was purified from pooled mature adipocytes in inguinal adipose tissue of wild-type and BMPR2 knockout mice. Each group had four mice. Uniquely indexed libraries were generated per sample with the NEBNext UltraTM RNA Library Prep Kit for Illumina (NEB,USA), according to the manufacturer’s instructions. Indexed libraries were sequenced using the Illumina Hiseq platform, generating 125 bp long paired-end reads, yielding a minimum of ~20 million total reads per sample. After assessing sequence quality, STAR was used to map reads to the mouse genome (mm10) with default setting values. Mapping efficiency was >95% in all experiments. Then mapped reads were annotated and FPKM (Fragments per kilo base per million mapped reads) was calculated with genecode M19 annotation reference. 10,435 protein coding genes with FPKM ≥ 1 were used for differential expression analysis with fold change 1.5 as cutoff. 576 genes were downregulated in BMPR2 adipocytes group. Downregulated genes were used for gene ontology analysis in KEGG pathways. Pathways with FDR < 0.1 and *p* value < 0.00015 were exported and visualized in Cytoscape v.3.4.0 in which the node sizes and color presented *p* value and FDR, respectively.

### Quantitative RT-PCR

Complementary DNA synthesized from total RNA was analyzed in a Sequence Detector (Q5; Bio-Rad) with specific primers and SYBR Green PCR Master reagents (ABI). The relative abundance of mRNAs was calculated with 18S mRNA as the invariant control. The primer sequences were from PrimerBank (http://pga.mgh.harvard.edu/primerbank/). The primers were synthesized by Sangon Biotech Company, Shanghai, China.

### Antibodies and immunoblotting

White adipose tissue and brown adipose tissue or cultured cells were homogenized in lysis buffer containing 2% SDS and 60 mM Tris.HCl (pH 6.8) and loaded onto the gel for electrophoresis. Proteins were then transferred onto nitrocellulose membrane and immunoblotted with specific antibodies. For determination of insulin stimulated P-AKT, iWAT was dissected from mice 5 min after peritoneal injection of insulin. Antibodies used included the following: BMPR2 (3F6F8) (Thermo, MA5-15827. 1:1000), Caspase 3 (Cell Signaling Technology (CST), 9662. 1:1000), cleaved caspase 3 (Asp175) (CST, 5A1E, 9664. 1:1000), caspase 9 (CST, 9508. 1:1000), cleaved caspase 9 (Asp353) (CST, 9509. 1:1000), cleaved caspase 1 (Asp296) (CST, 67314. 1:1000), p38/MAPK (Cell Signaling Technology, 9212. 1:1000), P-p38/MAPK (Cell Signaling Technology, 9216. 1:1000), perilipin (G-2) (Santa Cruz Biotechnology, sc-390169.1:500), Caspase 1 (abcam: ab138483, 1:1000), HSP90 (AC-16) (Santa Cruz Biotechnology, sc-101494. 1:500), and cytochrome C (D187C) (CST 11940, 1:1000).

### Statistics and reproducibility

All results are presented as means ± SD. Data were analyzed using a two-way ANOVA and a two-tailed Student’s *t* test was used to compare difference between WT and knockout group. Pearson correlation coefficient (R) was used as a measure of the linear correlation between expression of genes. A difference is considered significant as the following: *p < 0.05, **p < 0.01 and ***p < 0.001. There are 5–8 mice in each group in mouse experiments. Cell culture experiments were repeated at least three times.

### Reporting summary

Further information on research design is available in the Nature Research Reporting Summary linked to this article.

## Supplementary information

Supplementary Information

Supplementary Data 1

Description of Additional Supplementary Files

Reporting Summary

## Data Availability

The raw data of RNA-sequence used to generate the results shown in Fig. 4 and Supplementary Fig. 3 are available at Sequence Read Archive (SRA accession: PRJNA611934). The data used to generate the main results shown in Figs. 1–6 are available in Supplementary Data 1.
