# Supplementary material for: BMPR2 promotes fatty acid oxidation and protects white adipocytes from cell death in mice
Source: Commun Biol. 2020 Apr 29;3:200. doi: 10.1038/s42003-020-0928-y (PMC7190840; doi:10.1038/s42003-020-0928-y)
Supplement: Supplementary file 1 — Supplementary Information [file 42003_2020_928_MOESM1_ESM.pdf]

Supplemental information

Supplementary Figures and Figure Legends

Supplementary Figure 1

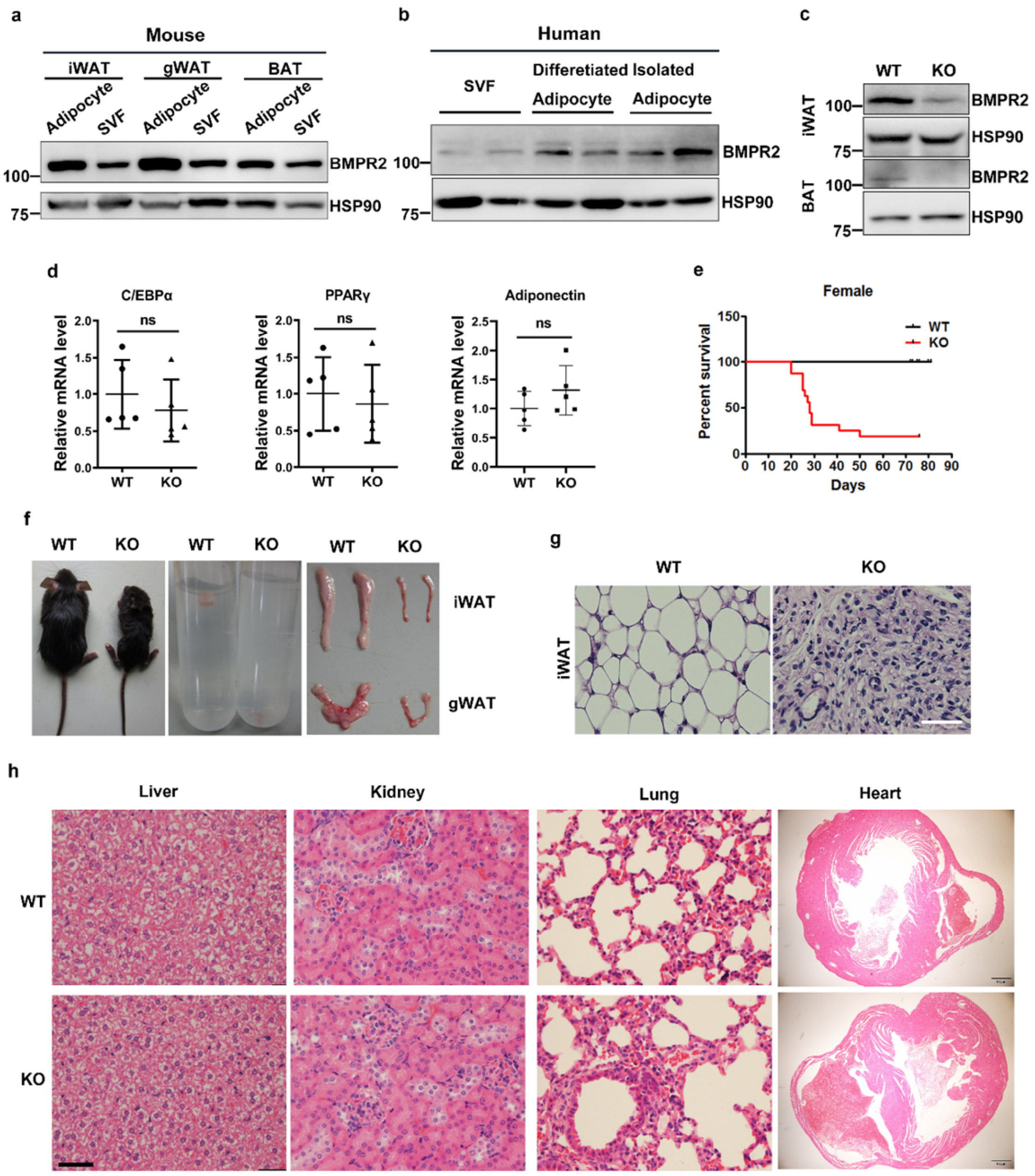

Supplementary Fig. 1: Phenotype of BMPR2 knockout mouse.

a: Western Blot for BMPR2 in adipocyte and stromal vascular fractions (SVF) of adipose tissue in 2 months old mouse.

b: Subcutaneous adipose tissue at hip region was obtained from human patient and enzymatically disassociated, SVF and mature adipocytes were collected, and a part of SVF was cultured and induced to differentiation, followed by Western Blot to determine BMPR2 level.

c: Western blot for BMPR2 in mature adipocytes isolated from iWAT and BAT of mice at 2 weeks old. Each lane represents sample prepared from pooled adipocytes of 4 mice.
d: Quantitative PCR data showing the relative expression level of two key genes of C/EBPα and PPARγ for adipogenic differentiation and one of adiponentcin for mature adipocyte in iWAT of male WT and KO mice at 2 weeks old (n = 5).
e: Survival curve of female BMPR2 knockout and WT mice.
f: Representative pictures of WT and KO female mice, their iWAT and gWAT, and iWAT in PBS. WT iWAT floats on water surface however that from KO mice sinks at the bottom of the tube.
g: H&E stained section of iWAT showing no visible adipocytes in male BMPR2 KO mice at 4 weeks old. Scale bar: 20μm.
h: H&E staining of several organs of mice at 19 days old for comparison of histological changes between two genotypes. Unmarked scale bar: 20μm.
Data are expressed as means ± SD. *p < 0.05, **p < 0.01, ***p < 0.001.

Supplementary Figure 2

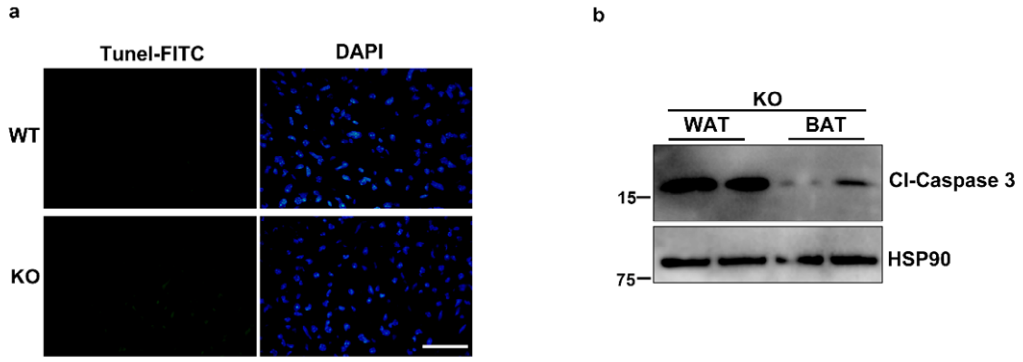

Supplementary Fig. 2: Character of WT and BMPR2 knockout adipose tissue.
a: Tunel assay analysis showing no positive cell in both BMPR2 knockout BAT of male mice at 3 weeks old. Scale bar: 20μm.
b: Western Blot for cleaved caspase 3 in WAT and BAT of male BMPR2 KO mice at 3 weeks old.

Supplementary Figure 3

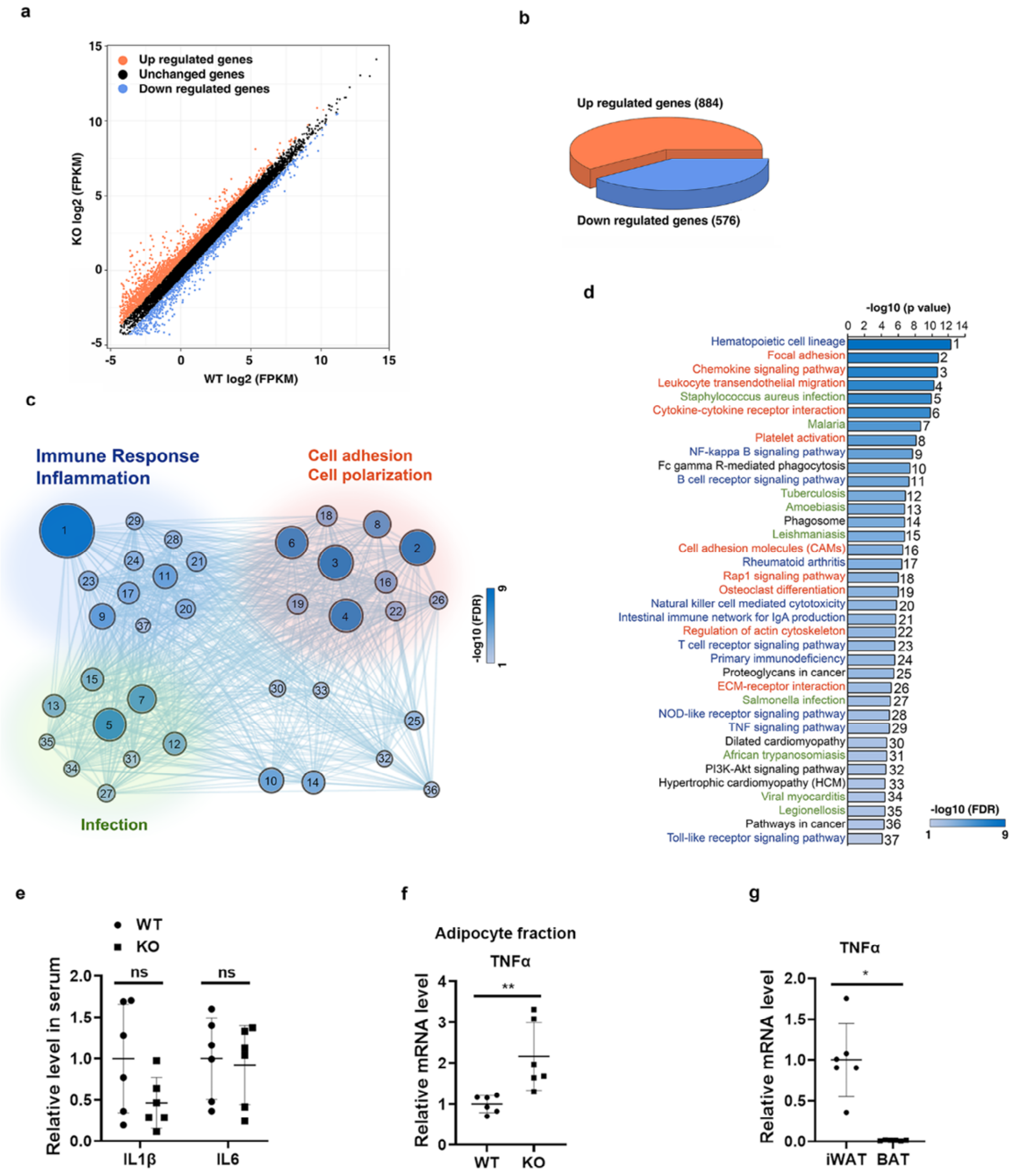

Supplementary Fig. 3: Inflammatory changes in adipose tissue at weaning.

a: XY plot shows differential expression of genes between WT and BMPR2 adipocytes. FPKM >= 1 with fold change 1.5 as cutoff

b: Pie plot shows the number of up and down regulated genes in BMPR2 knockout adipocytes.

c: Gene ontology analysis for up-regulated genes in KEGG pathways. Pathways with FDR <0.1 and p value < 0.00015 were visualized in Cytoscape v.3.4.0, the node sizes and color presented p-value and FDR, respectively.
d: Pathways with FDR <0.1 were listed and grouped designated with different color.
e: levels of IL1β and IL6 were determined with Elisa.
f: Quantitative PCR showed the relative mRNA level in adipocytes isolated from iWAT of mice at 14 days old. n=6
g: Quantitative PCR showed the relative mRNA level of TNFα in iWAT and BAT of mice at 3-week old. n=6
Data are expressed as means ± SD. *p < 0.05, **p < 0.01, ***p < 0.001.

Supplementary Figure 4

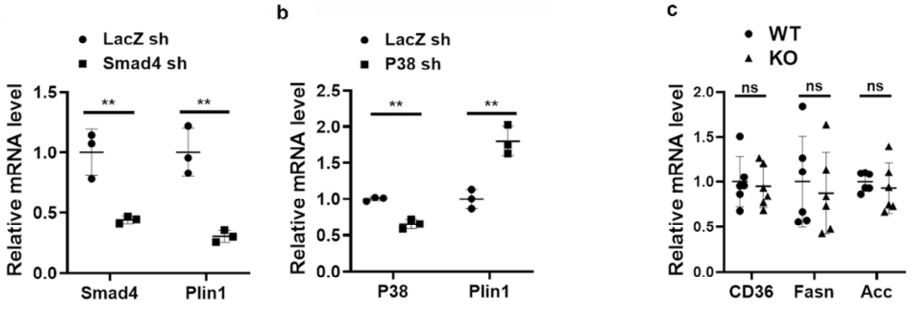

Supplementary Fig. 4: Gene expression of WT and BMPR2 KO adipocytes.
a: mRNA level of indicated genes in differentiated adipocytes from SVF with Smad4 shRNA bearing adenovirus infection.
b: mRNA level of indicated genes in differentiated adipocytes from SVF with P38 shRNA bearing adenovirus infection.
c: mRNA level of genes determined by realtime PCR in mature adipocytes isolated from inguinal fat from mice of 2 weeks old. n=6.
Data are expressed as means ± SD. *p < 0.05, **p < 0.01, ***p < 0.001.

Supplementary Figure 5

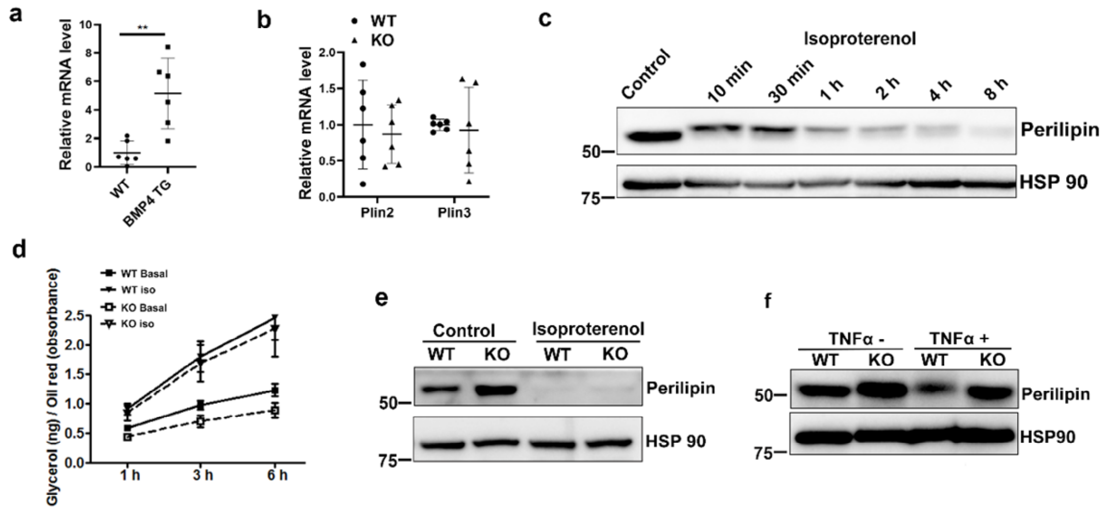

Supplementary Fig. 5: Regulation of perilipin while lipolysis.

a: Quantitative PCR analysis of plin1 in iWAT of BMP4 transgenic mice (FABP4 promoter) at 2 months old (n=6).

b: Quantitative PCR analysis of plin2 and plin3 in mature adipocytes isolated from iWAT at 2 weeks old (n=6).

c: Differentiated mature adipcytes were treated with 10 μM isoproterenol and Perilipin levels at different time were showed by Western Blot.

d: Glycerol in culture medium of cultured BMPR2 knockout and control adipocytes after 10 μM isoproterenol stimulation was determined by colorimetry and normalized to total amount of neutral lipid (stained Oil red O rinsed with methanol and absorbance measured at 490nm).

e: Perilipin determined by Western Blot in differentiated BMPR2 KO and WT adipocytes from precursors after treated with isoproterenol for 16 hours.

f: iWAT in 2 weeks old mice was minced into pieces of about 1m3 and treated with 50 ng/ml recombinant mouse TNFα for 16 hours, tissue lysate was subject to Western Blot to show perilipin level.

Data are expressed as means ± SD. *$p < 0.05$, **$p < 0.01$, ***$p < 0.001$.

Supplementary Figure 6

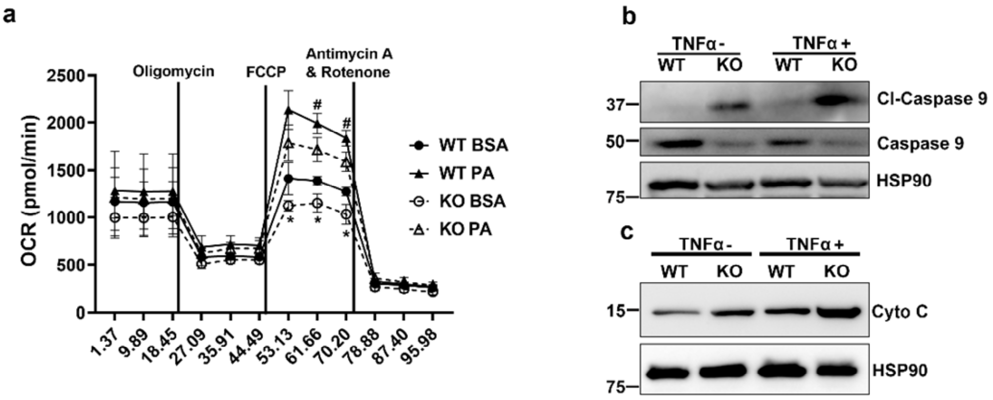

Supplementary Fig. 6: Inhibited lipolysis increases apoptotic susceptibility.

a: OCR determined using a seahorse XF24 analyzer. BSA: 0.17mM bovine serum albumin; PA: 2mM palmitate conjugated to 0.17mM BSA; The final concentration of the drugs for mitochondrial stress test are all 5μM; * $p < 0.05$ represents statistical significance between groups of WT BSA and KO BSA; # $p < 0.05$ represents statistical significance between groups of WT PA and KO PA.

b: Western Blot shows levels of Caspase 9 in BMPR2 KO and WT adipocytes with or without 20ng/ml TNFα for 16 hours.

c: Western Blot determined cytosolic cytochrome C (Cyto C) in adipocytes.

Supplementary Figure 7

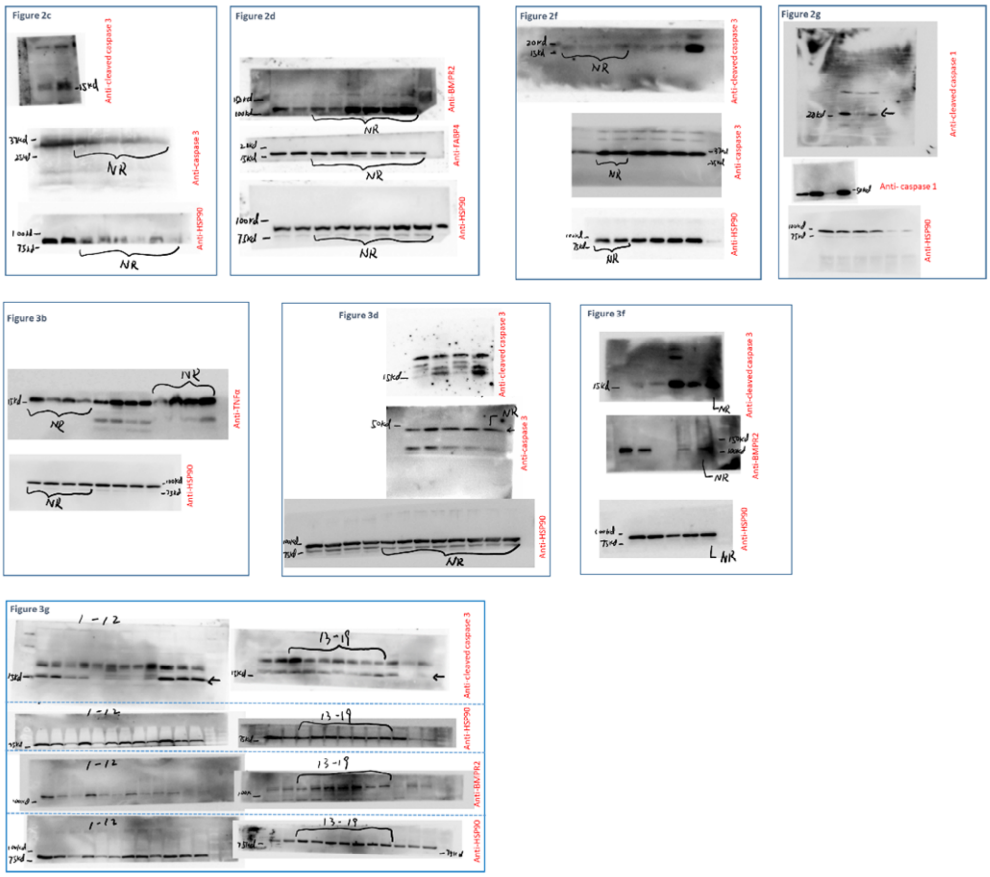

Supplementary Fig. 7: Original immunoblots for panels shown in Figures 2&3. 'NR' indicates lanes derived from non-relevant experimental samples.

Supplementary Figure 8

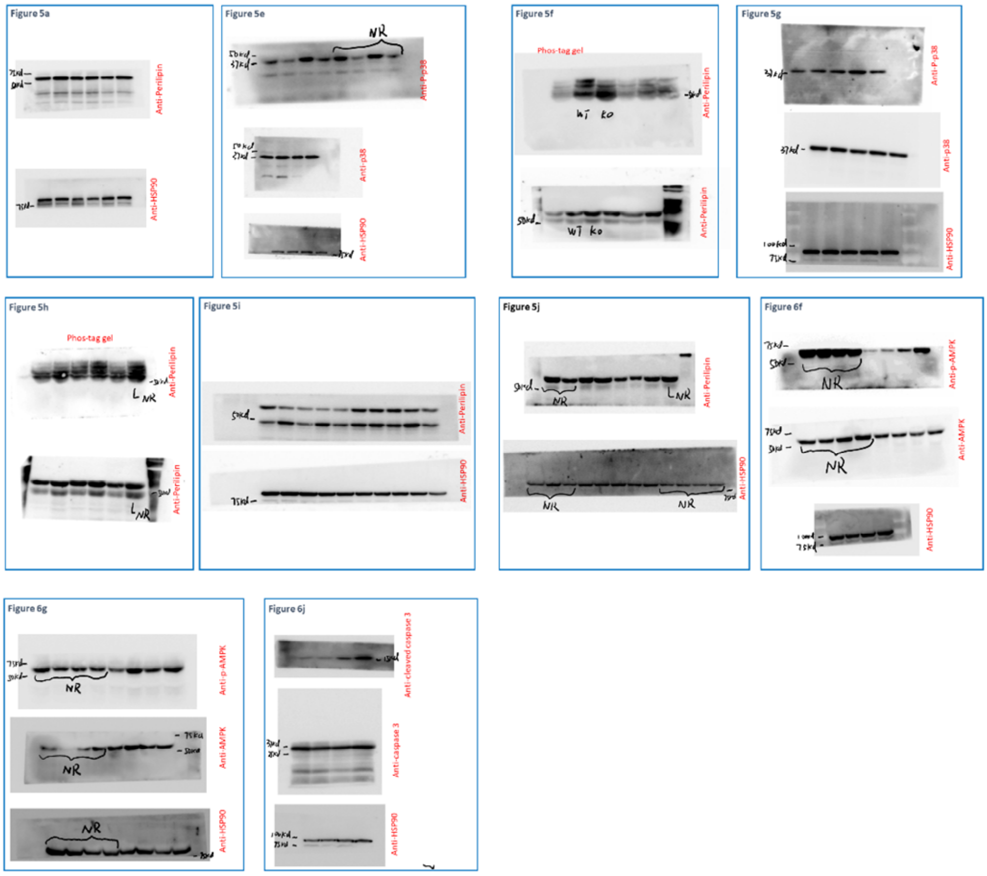

Supplementary Fig. 8: Original immunoblots for panels shown in Figures 5&6. ‘NR’ indicates lanes derived from non-relevant experimental samples.

Supplementary Figure 9

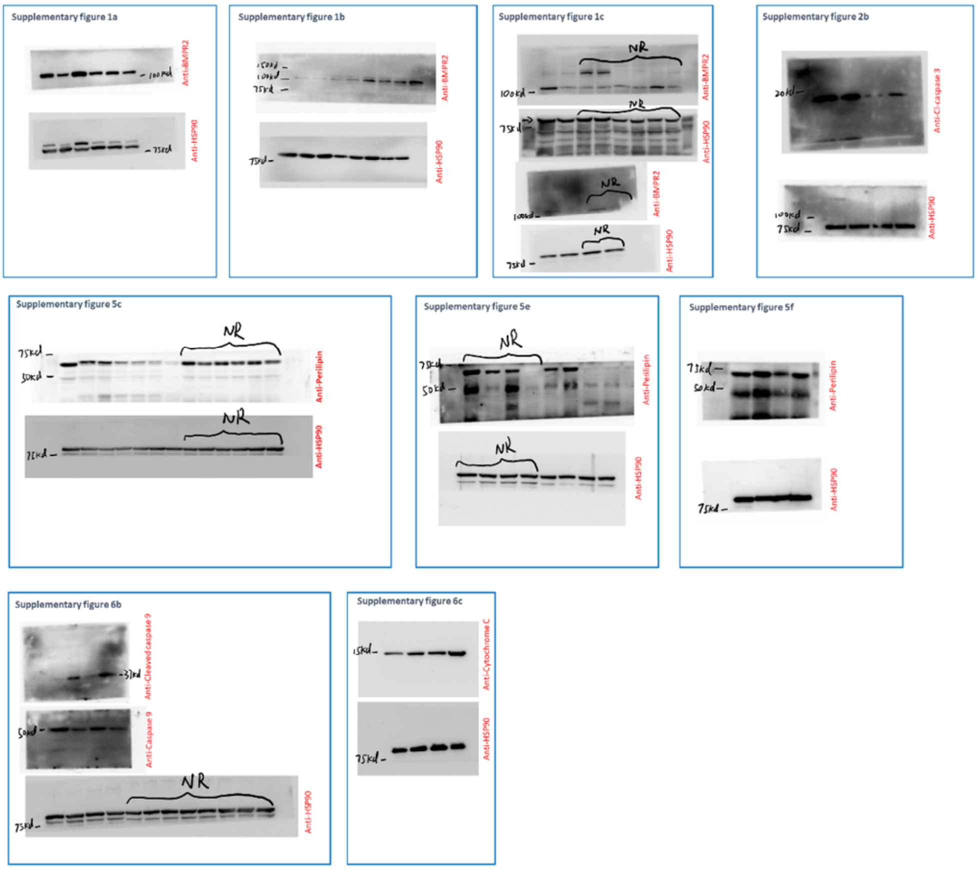

Supplementary Fig. 9: Original immunoblots for panels shown in Supplementary Figures 1, 2, 5, 6. 'NR' indicates lanes derived from non-relevant experimental samples.

Supplementary Table

Supplementary Table 1

Pathway term	FDR	Genes	
Oxidative phosphorylation	1.96372038968152E-48	Complex Ⅰ	NDUFAB1, NDUFS7, NDUFS6, NDUFS8, NDUFS3, NDUFS2, NDUFB11, NDUFB10, NDUFC2, NDUFA13, NDUFA10, NDUFA12, NDUFA11, NDUFB5, NDUFB6, NDUFB7, NDUFB8, NDUFB9, NDUFA4, NDUFA5, NDUFA3, NDUFA9, NDUFA6, NDUFV3, NDUFV1, NDUFV2
		Complex Ⅱ	SDHA, SDHB, SDHC, SDHD
		Complex Ⅲ	UQCRC2, UQCRC1, CYC1, UQCRFS1, UQCRQ, UQCR10, UQCR11
		Complex Ⅳ	COX5A, COX5B, COX4I1, COX6C, COX7B, COX7C, COX6B1, COX6B2, COX7A1, COX8B, COX8A
		Complex Ⅴ	ATP5D, ATP5B, ATP5L, ATP5O, ATP5H, ATP5K, ATP5C1, ATP5G2, ATP5J2, COX6A1, ATP5A1
Fatty acid metabolism	1.62119534463656E-06	ACAA2, CPT1B, SCD3, CPT2, ACADS, EHHADH, MCAT, ACACA, ECHS1, ACAT2, ACAT1, HADHA, HADHB, ACADVL, PECR, HADH, MECR	
Fatty acid degradation	0.0000684894967628579	ECI1, ACAA2, CPT1B, GCDH, CPT2, ACADS, EHHADH, ECHS1, ACAT2, ACAT1, HADHA, HADHB, ACADVL, ADH1, HADH	
Fatty acid elongation	0.000248012669912256	ACAA2, ELOVL3, ACOT2, ECHS1, ACOT1, HADH, MECR, HADHA, ACOT4, HADHB, ACOT3	
Peroxisome	2.51056927469406E-06	HACL1, PAOX, ECH1, EHHADH, AMACR, PEX6, CRAT, DDO, PIPOX, SOD2, PEX11A, PECR, DHRS4, MLYCD, MPV17L, PEX26, NUDT7, PEX16, IDH2, PXMP2, SLC27A2	
PPAR signaling pathway	0.0954838769012367	CPT1B, PPARA, SCD3, CPT2, EHHADH, RXRG, UCP1, PCK1, APOA1, CYP27A1, PLIN1, APOC3, FABP3, SLC27A2	

Supplementary table 1: Differentially expressed genes involving lipid metabolism and oxidative phosphorylation pathways.
