# Supplementary material for: BMPR2 promotes fatty acid oxidation and protects white adipocytes from cell death in mice
Source: Commun Biol. 2020 Apr 29;3:200. doi: 10.1038/s42003-020-0928-y (PMC7190840; doi:10.1038/s42003-020-0928-y)
Supplement: Supplementary file 3 — Description of Additional Supplementary Files [file 42003_2020_928_MOESM3_ESM.pdf]

Description of Additional Supplementary Files

File Name: **Supplementary Data 1**

Description: The Supplementary Data 1 file contains all source data underlying the graphs and charts presented in the main figures (Figure 1-6). The source data includes relative mRNA expressions, body weight, tissue weight, cell diameter, serum concentration of glucose and triglyceride, quantification of positive staining area of IHC, relative grey density of bands of Western blot, relative released glycerol from cultured cells, and value of OCR of seahorse assay.
