# Supplementary material for: BMPR2 promotes fatty acid oxidation and protects white adipocytes from cell death in mice
Source: Commun Biol. 2020 Apr 29;3:200. doi: 10.1038/s42003-020-0928-y (PMC7190840; doi:10.1038/s42003-020-0928-y)
Supplement: Supplementary file 4 — Reporting Summary [file 42003_2020_928_MOESM4_ESM.pdf]

nature research

Corresponding author(s): Qi-qun Tang

Last updated by author(s): Apr 1, 2020

Reporting Summary

Nature Research wishes to improve the reproducibility of the work that we publish. This form provides structure for consistency and transparency in reporting. For further information on Nature Research policies, see Authors & Referees and the Editorial Policy Checklist.

Statistics

For all statistical analyses, confirm that the following items are present in the figure legend, table legend, main text, or Methods section.

n/a	Confirmed	
☐	☒	The exact sample size (*n*) for each experimental group/condition, given as a discrete number and unit of measurement
☐	☒	A statement on whether measurements were taken from distinct samples or whether the same sample was measured repeatedly
☐	☒	The statistical test(s) used AND whether they are one- or two-sided<br>*Only common tests should be described solely by name; describe more complex techniques in the Methods section.*
☒	☐	A description of all covariates tested
☒	☐	A description of any assumptions or corrections, such as tests of normality and adjustment for multiple comparisons
☐	☒	A full description of the statistical parameters including central tendency (e.g. means) or other basic estimates (e.g. regression coefficient) AND variation (e.g. standard deviation) or associated estimates of uncertainty (e.g. confidence intervals)
☐	☒	For null hypothesis testing, the test statistic (e.g. *F*, *t*, *r*) with confidence intervals, effect sizes, degrees of freedom and *P* value noted<br>*Give P values as exact values whenever suitable.*
☒	☐	For Bayesian analysis, information on the choice of priors and Markov chain Monte Carlo settings
☒	☐	For hierarchical and complex designs, identification of the appropriate level for tests and full reporting of outcomes
☒	☐	Estimates of effect sizes (e.g. Cohen's *d*, Pearson's *r*), indicating how they were calculated

Our web collection on statistics for biologists contains articles on many of the points above.

Software and code

Policy information about availability of computer code

Data collection	Zen data acquisition and analysis
Data analysis	Graphpad Prism, statistics and graphing program, Image J

For manuscripts utilizing custom algorithms or software that are central to the research but not yet described in published literature, software must be made available to editors/reviewers. We strongly encourage code deposition in a community repository (e.g. GitHub). See the Nature Research guidelines for submitting code & software for further information.

Data

Policy information about availability of data

All manuscripts must include a data availability statement. This statement should provide the following information, where applicable:

- Accession codes, unique identifiers, or web links for publicly available datasets
- A list of figures that have associated raw data
- A description of any restrictions on data availability

The authors affirm that the data that support the findings of this study are found within the paper or in Supplementary Information and Supplementary Data 1.

Field-specific reporting

Please select the one below that is the best fit for your research. If you are not sure, read the appropriate sections before making your selection.

☒ Life sciences ☐ Behavioural & social sciences ☐ Ecological, evolutionary & environmental sciences

For a reference copy of the document with all sections, see nature.com/documents/nr-reporting-summary-flat.pdf

Life sciences study design

All studies must disclose on these points even when the disclosure is negative.

Sample size	Mouse experiments: 5-8 in each group. Cell culture experiments : Repeated at least 3 times.
Data exclusions	No data was excluded from the analysis.
Replication	Biological repeats were performed for each study. For cell culture experiments, technical repeats were performed for each individual experiment.
Randomization	No randomization of mice. Mice analyzed were litter mates.
Blinding	Investigators were not blinded to mouse genotypes during experiments.

Reporting for specific materials, systems and methods

We require information from authors about some types of materials, experimental systems and methods used in many studies. Here, indicate whether each material, system or method listed is relevant to your study. If you are not sure if a list item applies to your research, read the appropriate section before selecting a response.

Materials & experimental systems

n/a	Involved in the study	
☐	☒	Antibodies
☒	☐	Eukaryotic cell lines
☒	☐	Palaeontology
☐	☒	Animals and other organisms
☐	☒	Human research participants
☒	☐	Clinical data

Methods

n/a	Involved in the study	
☒	☐	ChIP-seq
☒	☐	Flow cytometry
☒	☐	MRI-based neuroimaging

Antibodies

Antibodies used	All the antibodies are commercial available, and the name of supplier and cataloger number are described in method.
Validation	Validation is available on the manufacturer's website for use in mouse and human tissue.

Animals and other organisms

Policy information about studies involving animals; ARRIVE guidelines recommended for reporting animal research

Laboratory animals	All the mice have been crossed back to C57/B6L for more than six generations. Animals are male mice unless otherwise stated. Age of the mice are discribed in the figure legend of each experiment.
Wild animals	Not applicable
Field-collected samples	Not applicable
Ethics oversight	All studies were approved by the Animal Care and Use Committee of Shanghai Medical College, Fudan University.

Note that full information on the approval of the study protocol must also be provided in the manuscript.

Human research participants

Policy information about studies involving human research participants

Population characteristics	Patients underwent plastic surgery irrelevant to metabolic disease in Shanghai Jiaotong University Affiliated Sixth People's Hospital.
Recruitment	Patients were both male and female, aged between 25 and 60 years old, and had no diagnostic metabolic disorders.
Ethics oversight	This study was approved by the ethics committees of Shanghai Medical College, Fudan University.

Note that full information on the approval of the study protocol must also be provided in the manuscript.
